# Bioinformatic analysis reveals the association between bacterial morphology and antibiotic resistance using light microscopy with deep learning

**DOI:** 10.3389/fmicb.2024.1450804

**Published:** 2024-09-19

**Authors:** Miki Ikebe, Kota Aoki, Mitsuko Hayashi-Nishino, Chikara Furusawa, Kunihiko Nishino

**Affiliations:** ^1^SANKEN (Institute of Scientific and Industrial Research), Osaka University, Osaka, Japan; ^2^Graduate School of Pharmaceutical Sciences, Osaka University, Suita, Japan; ^3^Artificial Intelligence Research Center (AIRC-SANKEN), Osaka University, Osaka, Japan; ^4^Center for Biosystems Dynamics Research, RIKEN, Suita, Japan; ^5^Universal Biology Institute, The University of Tokyo, Tokyo, Japan; ^6^Center for Infectious Disease Education and Research, Osaka University, Osaka, Japan

**Keywords:** antibiotic resistance, light microscopy, bacterial morphology, deep learning, bioinformatic analysis

## Abstract

Although it is well known that the morphology of Gram-negative rods changes on exposure to antibiotics, the morphology of antibiotic-resistant bacteria in the absence of antibiotics has not been widely investigated. Here, we studied the morphologies of 10 antibiotic-resistant strains of *Escherichia coli* and used bioinformatics tools to classify the resistant cells under light microscopy in the absence of antibiotics. The antibiotic-resistant strains showed differences in morphology from the sensitive parental strain, and the differences were most prominent in the quinolone-and β-lactam-resistant bacteria. A cluster analysis revealed increased proportions of fatter or shorter cells in the antibiotic-resistant strains. A correlation analysis of morphological features and gene expression suggested that genes related to energy metabolism and antibiotic resistance were highly correlated with the morphological characteristics of the resistant strains. Our newly proposed deep learning method for single-cell classification achieved a high level of performance in classifying quinolone-and β-lactam-resistant strains.

## Introduction

The emergence of multidrug-resistant bacteria that survive in the presence of multiple types of antibiotics is a global problem, and the spread of these bacterial strains is becoming a threat to public health. Drug resistance is caused primarily by long-term overuse of antibacterial medications. The factors and molecular mechanisms responsible for drug resistance in microbes have been widely reported (Alekshun and Levy, 2007). Accordingly, it has long been known that bacterial cell morphology alters following exposure to antibiotics, with cells undergoing filamentation in response to this stress (Nishino and Nakazawa, 1972; Elliott et al., 1987). In recent years, the effects of antibiotics on bacterial morphology have been studied in terms of bacterial adaptation and survival in response to drug treatments (Monahan et al., 2014; Banerjee et al., 2021). However, the morphology of antibiotic-resistant bacteria in the absence of drugs is not well known.

Laboratory-based evolution is a powerful tool for investigating the dynamics of acquiring drug resistance (Suzuki et al., 2014; Furusawa et al., 2018; Maeda et al., 2020). Using this technique, bacterial cells are exposed to fixed concentrations of drugs, around which cell growth is partially or completely inhibited such that a selective advantage for resistant strains is maintained. Suzuki et al. (2014) performed laboratory-based evolution experiments using *Escherichia coli* under long-term treatment with various antibiotics to obtain resistant strains. Ten antibiotic-resistant strains were identified and transcriptome and genome sequencing analyses were performed to identify gene expression changes and fixed mutations. Because many gene expression changes were observed in the antibiotic-resistant strains, it was hypothesized that these changes may affect bacterial morphology (Suzuki et al., 2014). Using these resistant strains, our laboratory previously reported significant morphological differences between an enoxacin-resistant strain compared with the antibiotic-sensitive parental strain in the absence of the drug, with the changes in cell structure being accurately discernible using deep learning of electron microscopy images (Hayashi-Nishino et al., 2022).

The objectives of the present study were to elucidate the morphological characteristics of the 10 antibiotic-resistant *E. coli* strains using bioinformatics tools and identify the genetic influences on the morphology of the resistant strains. Light microscopy images were used because they are much easier and faster to obtain than electron microscopy images and suitable for analyzing large numbers of living bacterial cells. Moreover, we aimed to discern antibiotic-resistant strains from the antibiotic-sensitive parental strain in the absence of drugs using a newly proposed cell contour-based deep learning method.

## Materials and methods

### Bacterial strains and culture conditions

Ten laboratory-evolved antibiotic-resistant *E. coli* strains and their parental MDS42 strain (Table 1) (Suzuki et al., 2014) were used in the experiments. A single colony of each resistant strain was firstly obtained from the above original resistant strains described in the following section. Modified M9 medium (Mori et al., 2011) were prepared as described in the Supplementary methods. For morphological observations, bacterial strains were cultured as described previously (Hayashi-Nishino et al., 2022). Briefly, the antibiotic-resistant and parental strains were precultured in M9 medium at 34°C for 23 h with shaking at 432 rpm in Nunc 96-well microplates (Thermo Fisher Scientific Inc.). The cells were then diluted to an optical density (OD)_600 nm_ of 1 × 10^−4^ to 1 × 10^−8^ in 5.0 mL of fresh M9 medium in glass test tubes and further incubated with shaking at 150 rpm in a water bath (TAITEC Corp.) at 34°C until the cultures reached an OD_600 nm_ in the range of 0.07–0.13, the same range used by Suzuki et al. (2014) for RNA isolation for gene expression analysis. The final OD_600 nm_ values were determined from 200 μL aliquots of the culture transferred to a Nunc 96-well microplate.

**Table 1:** List of the antibiotic-resistant bacterial strains used in this study.^a^

Antibiotic-resistant strains	Antibiotic name	Class	Cellular target
CPZ	Cefoperazone	Cephalosporin, β-lactam (BL)	Cell wall
CFIX	Cefixime	Cephalosporin, β-lactam (BL)	Cell wall
AMK	Amikacin	Aminoglycoside (AG)	Protein synthesis, 30S
NM	Neomycin	Aminoglycoside (AG)	Protein synthesis, 30S
DOXY	Doxycycline	Tetracycline (TC)	Protein synthesis, 30S
CP	Chloramphenicol		Protein synthesis, 50S
AZM	Azithromycin	Azalide, macrolide (ML)	Protein synthesis, 50S
TP	Trimethoprim		Folic acid synthesis
ENX	Enoxacin	Quinolone (QN)	DNA gyrase
CPFX	Ciprofloxacin	Quinolone (QN)	DNA gyrase

a The name of the antibiotic-resistant strains corresponds to the abbreviation of the antibiotics used in the bacterial evolution experiment reported previously (Suzuki et al., 2014).

### Single colony isolation and determination of minimum inhibitory concentrations

Serial dilutions of each antibiotic were made in 96-well microplates (Thermo Fisher Scientific Inc.) using modified M9 medium and stored at −80°C before use. The range of antibiotic concentrations used for minimum inhibitory concentrations (MICs) was based on two-fold dilution steps up and down from 1 μg/mL, as required depending on the antibiotic (Suzuki et al., 2014). Single colony isolation and determination of MICs were performed as follows:

1. Each resistant strain was cultured on modified M9 agar plates (Supplementary methods) (Mori et al., 2011) at 34°C for two days.
2. Three colonies were chosen and suspended in modified M9 medium (Mori et al., 2011) to yield an initial OD_600 nm_ of 3 × 10^−5^. This suspension was then inoculated into each well of freshly thawed MIC plates to a final volume of 200 μL. The plates were incubated at 34°C for 23 h with shaking 432 rpm on a multimode microplate reader (Infinite M200 PRO, TECAN Ltd.).
3. The OD_600 nm_ of each well was measured with a microplate reader, and the well with the highest antibiotic concentration that had an OD_600 nm_ > 0.03 was chosen for further MIC determination. A portion of the culture from the selected well was stored in modified M9 medium containing 15% glycerol at −80°C, and the remaining culture was used for MIC measurement.
4. The remaining cell cultures were diluted in M9 medium to yield an initial OD_600 nm_ of 3 × 10^−5^ and inoculated into each well of freshly thawed MIC plates to a final volume of 200 μL. The plates were incubated at 34°C for 23 h with shaking on a multimode microplate reader. The OD_600 nm_ of each well was measured, and the lowest antibiotic concentration that reduced the growth to an OD_600 nm_ < 0.03 was defined as the MIC.
5. The MICs of the parental and the original resistant strains (Suzuki et al., 2014) were determined as described above. The relative MIC log_2_ values were calculated by comparing the MIC values of the original resistant strain to those of the parental strain, and by comparing the MIC values of colonies isolated from the original resistant strain to those of the parental strain. The colony with the MIC log_2_ value closest to that of the original resistant strain was selected and used for further experiments.

### Image acquisition

Bacterial cell cultures were centrifuged, and the resulting cell pellets were resuspended in phosphate buffered saline (PBS, Sigma-Aldrich) and washed twice. The cell pellets were suspended in 20 μL of PBS and the suspensions were further diluted to a ratio of 1:10 in PBS. Then, 1.2 μL of the cell suspension were mounted on a glass slide and covered with a 22 × 22 mm cover slip (Matsunami, Japan). A phase contrast microscope with a 100× objective lens (Leica Microsystems) was used for observations. Microscopy images were captured using a Leica ICC50 W camera with LAS EZ imaging software (v. 3.4) at a resolution of 96 pixels/inch. Single images were obtained at a resolution of 2,592 pixels × 1,944 pixels in *xy*. The exposure time was 123 ms, with a gain value of 1.0×, a gamma value of 0.60, and a brightness value of 1.0. The images were saved as tiff files.

Microscopy image data were obtained from each antibiotic-resistant strain, which comprised four lines and the parental strain as one set of data and collected three datasets from bacteria cultured on different dates in each set. Approximately 20 images of each bacterial specimen were taken and used for analysis.

### Segmentation and feature extraction from cells

As a preprocessing step, denoising of each image was carried out using a Gaussian filter (σ = 4). Cellular segmentation was then performed using Omnipose v. 0.4.4^1^ (Cutler et al., 2022) pretrained for bacterial phase contrast images. Postprocessing involved removing small regions (<96 pixels) to exclude cases of segmentation failures and remnants of dead bacteria, and to fill holes as much as possible.

After segmentation, the following 10 morphological parameters from each region were measured: Area, perimeter (Perim), Major, Minor, circularity (Circ), maximum Feret’s diameter (MaxFeret), minimum Feret’s diameter (MinFeret), aspect ratio (AR), roundness (Round), and solidity (Solid) (see Table 2 for definitions these features). The upper and lower 1% of the measured parameters were considered outliers and removed from the analysis.

**Table 2:** Morphological parameters measured in each bacterial strain.^a^

Parameter	Abbreviation	Unit	Definition
Area	–	μm^2^	Area
Perimeter	Perim	μm	The length of the outside boundary
Major	–	μm	Primary axis of the best fitting ellipse
Minor	–	μm	Secondary axis of the best fitting ellipse
Circularity	Circ	–	4π × Area/(Perimeter)^2^ (A value of 1.0 indicates a perfect circle)
Maximum Feret’s diameter	MaxFeret	μm	The longest distance between any two points along the boundary
Minimum Feret’s diameter	MinFeret	μm	The shortest distance between any two points along the boundary
Aspect ratio	AR	–	Major/minor
Roundness	Round	–	The inverse of aspect ratio
Solidity	Solid	–	Area/convex area

a Ten parameters were measured in each strain of bacteria.

### Histogram intersection

Histogram intersections (Swain and Ballard, 1991) were used to examine the similarity between the parental strain and each resistant strain, or between resistant strains, and to examine reproducibility over three experiments. In each histogram, the range of values was divided into 100 parts, or 100 bins, and normalized so that the sum of all bins was equal to 1. The histogram intersection between two histograms $h_{1}$ and $h_{2}$ is then calculated as follows:


$$d\left(h_{1},h_{2}\right)=\sum_{i=1}^{100}\min\left(h_{1}\left(i\right),h_{2}\left(i\right)\right)$$

### Cluster analysis

A k-means clustering method was adopted to group bacterial cells into clusters according to the 10 abovementioned morphological features so that the cells in each cluster had similar morphological characteristics. The morphological features were standardized so that the mean and the standard deviation for each feature were 0 and 1, respectively, in advance of clustering. A principal component analysis (PCA) of each cluster was conducted, and the results were represented as a biplot depicting both the distribution of samples from each bacterial strain as an ellipse (a normal distribution) and the loading of each feature as an arrow. The biplots can be interpreted as follows: (1) distributions that are close to each other have similar features; (2) features that point in similar directions are highly correlated; and (3) a distribution that is on the same side as a given feature highly contributes to it. Furthermore, to display the representative shape of each cluster of cells, a sequence of contour points representing a cell was evenly interpolated so that the number of points was the same for all contours and then the mean and standard deviation of the coordinates of the points were calculated for each cluster.

### Weighted gene correlation network analysis

A network coexpression analysis was performed using the WGCNA R package v.1.72–1 (R v.4.3.0) (Langfelder and Horvath, 2008) to determine correlations between gene expression (Suzuki et al., 2014) and the morphological features of cells cultured as described above and observed in this study. The transcriptome data of resistant strains obtained by Suzuki et al. (2014) were utilized for the WGCNA. The dataset included 2,829 genes with expression levels exceeding a log-transformed threshold of 300 to account for background noise, as described in Suzuki et al. (2014). During data cleaning, the gene *rrsG*, which exhibited the same expression levels across all strains, was eliminated. First, a coexpression network was constructed wherein the nodes corresponded to gene expression profiles and the edges between genes were determined by the absolute value of the correlation coefficient, with soft-thresholding between the nodes as follows:


$$a_{ij}={\left|s_{ij}\right|}^{\beta },s_{ij}=\left|cor\left(x_{i},x_{j}\right)\right|$$

where $x_{i}$ is the i-th gene expression profile and β is the soft-thresholding parameter. A scale-free topology analysis was applied to choose an appropriate soft-thresholding power. Then, modules were identified as clusters of highly interconnected genes by hierarchical clustering with an average linkage method and the Dynamic Tree Cut method on the basis of interconnectedness defined by the topological overlap measure with a minimum cluster size of 30, a deep split of 2, and no respect of dendrogram. Those modules that were closely related with each other were merged according to a correlation threshold of 0.25. The gene expression profiles of each module were summarized by an eigengene that was defined as the first principal component of the expression matrix. Finally, the modules (genes) most correlated with each morphological feature were identified for further analysis.

### Gene ontology annotation and enrichment analysis

Genes found using the WGCNA were annotated according to the EcoCyc (Keseler et al., 2021) and Kyoto Encyclopedia of Genes and Genomes (KEGG) databases (Kanehisa et al., 2016). Then, gene ontology (GO)-term annotation and enrichment analyses were performed using the PANTHER classification system (v.18.0^2^), operated by the GO Consortium, which provides the largest free biological databases for a variety of species (Ashburner et al., 2000; Thomas et al., 2022; Aleksander et al., 2023). The parameters used were as follows: analysis type: PANTHER Overrepresentation Test (Released 20231017); annotation version: GO database DOI: 10.5281/zenodo.7942786 (Released 20230105); reference list: *Escherichia coli* (all genes in database); test type: Fisher’s exact. Multiple testing was corrected by calculating the false discovery rate (FDR) and FDR *p* < 0.05 was considered statistically significant.

### Deep neural networks for cell classification

For classification of single cells between the parental strain and each resistant strain, we developed a deep neural network, taking a sequence of cell contour points as the input. Cell regions were segmented from microscopy images by Omnipose as described above. A sequence of the coordinates of the contour points was then extracted from the segmented region of each single-cell and aligned so that the major axis was horizontal. The number of contour points differs among single cells depending on their sizes and shapes and should be the same as the input to the contour-based classifier models. An aligned sequence of the coordinates of contour points, therefore, was linearly interpolated on an equally spaced grid. The number of contour points was set to 128 in the following experiments.

The Residual Network (ResNet) architecture (He et al., 2016) incorporates shortcut connections to allow deeper networks without the degradation of training accuracy and is widely adopted as a backbone for state-of-the-art neural networks. Circular convolution (Peng et al., 2020) was proposed to extract effective features from object boundaries and mainly applied for instance segmentation and object detection. We integrated circular convolution layers into the ResNet architecture to learn the discriminative features of bacterial cell morphology in an end-to-end fashion. A circular convolution layer was created using the coordinates/features of neighboring points as the input, and the convolution operation with a one-dimensional kernel was performed, sliding all the way around a cell contour, to produce outputs representing the morphological properties of the bacterial cells.

Classifier models were trained from scratch for 100 epochs using a stochastic gradient descent algorithm (LeCun et al., 2012) with a weight decay of 0.0001, a momentum of 0.9, and a batch size of 128. The initial learning rate was set to 0.0001 and decreased following the cosine schedule (Loshchilov and Hutter, 2016). Data augmentation was conducted by adding a Gaussian noise with a sigma value of 0.01 to the coordinates of the contour points, smoothing by the Savitzky–Golay filter (Savitzky and Golay, 1964) with a window length of 5 and a polynomial order of 2, and randomly shifting the starting point.

Experiments were conducted using threefold cross-validation—because the microscopic image acquisition procedures were repeated three times, two of three datasets were used as a training set and the remaining dataset served as a test set. The classification performance of our proposed method was evaluated by the mean and standard deviation of the area under the receiver operating characteristic curve (AUC), sensitivity, and specificity over three datasets. Sensitivity, specificity, and accuracy were defined as follows:


(1) $$\text{Sensitivity}=\frac{TP}{TP+FP}$$

(2) $$\text{Specificity}=\frac{TN}{TN+FN}$$

(3) $$\text{Accuracy}=\frac{TP+TN}{TP+FP+TN+FN}$$

where TP denotes true positives (correctly classified resistant cells), FP denotes false positives (parental cells that were classified as resistant cells), TN denotes true negatives (correctly classified parental cells), and FN denotes false negatives (resistant cells that were classified as parental cells), respectively. We also conducted experiments to discriminate patches extracted from the microscopy images to compare our proposed approach with the original ResNet model with respect to its performance in classifying the parental strain and each resistant strain. A patch (224 × 224 pixels) was extracted from the center of a cell region and discarded if the area overlapping with another patch, which was evaluated by the intersection over union, was greater than 0.5 (50 percent). The intersection over union (IoU) is defined as follows:


$$IoU=\frac{\left|A\cap B\right|}{\left|A\cup B\right|}$$

where *A* and *B* are the areas of two patches, the denominator represents the area of union, and the numerator represents the area of intersection, respectively. Data augmentation was conducted by randomly flipping and rotating patches.

## Results

### Morphological variations exhibited by antibiotic-resistant *Escherichia coli* strains

Single colonies were isolated from 10 strains of antibiotic-resistant *E. coli*, as listed in Table 1, for microscopy-based observation of cell morphology. Most isolates, comprised of four lines from each resistant strain, exhibited resistance in accordance with the original strain (Suzuki et al., 2014) (Supplementary Figure S1). These isolates were then cultured in the absence of antibiotics and observed under phase contrast light microscopy.

**Figure 1:**
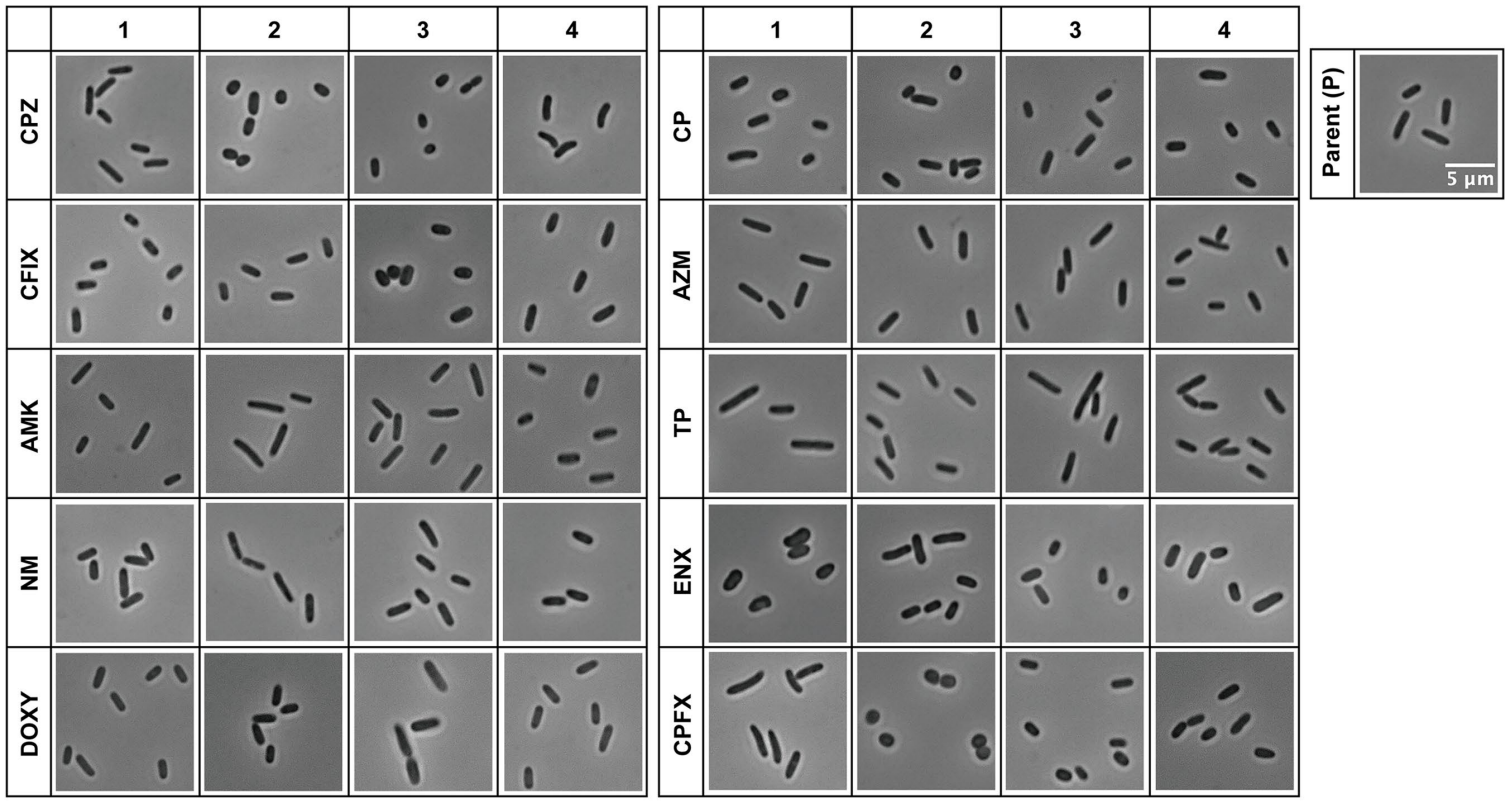
Light microscopy of antibiotic-resistant strains. Representative images of antibiotic-resistant *Escherichia coli* strains and the sensitive parental strain (P) from one of three datasets (Dataset 1) are shown. Four lines were isolated from each resistant strain, and the numbers at the top indicate the line of the resistant strain. The scale bar shown in the image of the parental strain applies to all panels. AMK, Amikacin; AZM, Azithromycin; CFIX, Cefixime; CP, Chloramphenicol; CPFX, Ciprofloxacin; CPZ, Cefoperazone; DOXY, Doxycycline; ENX, Enoxacin; NM, Neomycin; TP, Trimethoprim.

The resistant strains showed a variety of morphologies that differed from the structure of the rod-shaped, sensitive, parental strain (Figure 1). For example, CPZ-, CFIX-, and CP-resistant cells appeared shorter or smaller than the parental cells, whereas the ENX-and CPFX-resistant cells exhibited rounder or fatter morphology compared to the parental cells, in agreement with our previous study (Hayashi-Nishino et al., 2022). On the contrary, some of the TP-and AMK-resistant cells showed slightly elongated morphology, although this varied among the four lines. The majority of the antibiotic-resistant strains showed different cell morphologies from the parental strain; however, the NM-, DOXY-, and AZM-resistant strains were difficult to evaluate qualitatively. We therefore proceeded with a quantitative evaluation of the morphologies of the resistant and parental strains.

### Quantitative differences in cell morphology between the parental and antibiotic-resistant strains

A morphometric analysis was performed to evaluate the quantitative differences in cell morphology between the antibiotic-resistant and parental strains. We obtained microscopy image data from each resistant strain and the parental strain as one set of data and collected three datasets from bacteria cultured on different dates in each set to evaluate data variance caused by differences in the experiment. Then, single cells were extracted from the microscopy images using Omnipose, a CNN-based image segmentation tool applicable to various bacterial species and morphologies (Cutler et al., 2022). Examples of segmented resistant and parental cells are provided in Supplementary Figure S2. Several thousands of cells were segmented from each bacterial strain in a single dataset. These segmented cells were collected from the three datasets and used for analysis (Supplementary Table S1). Then, 10 morphological parameters (Area, Perim, Major, Minor, Circ, MaxFeret, MinFeret, AR, Round, and Solid) were measured from the extracted contours of the cells.

**Figure 2:**
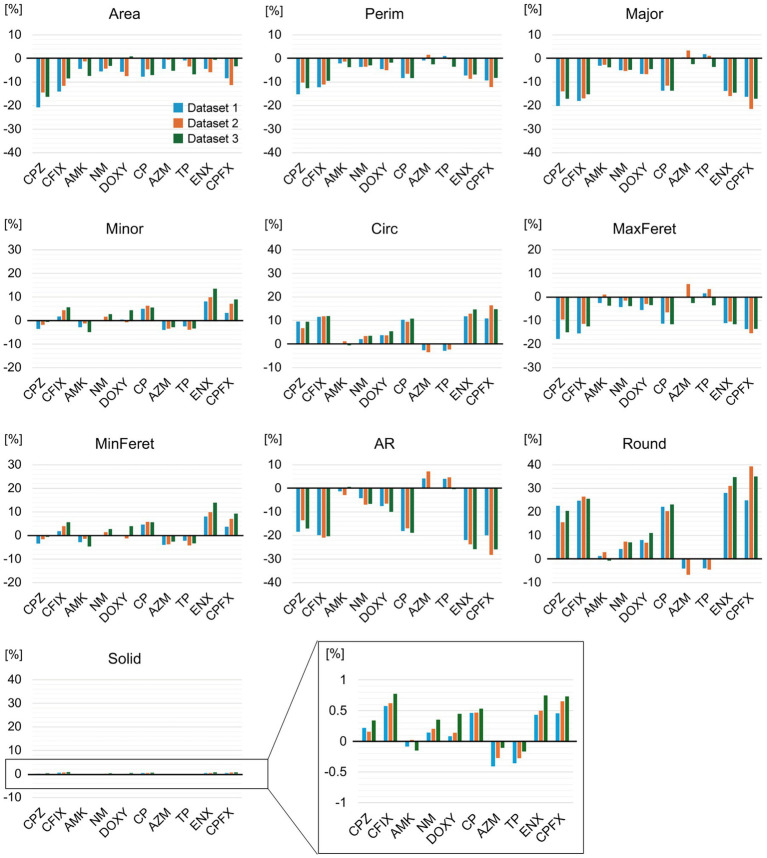
Changes in morphology between the parental and antibiotic-resistant strains. The change ratios of the median values for each morphological feature of the indicated drug-resistant strain against the parental strain are shown. The vertical axis represents the percentage change [%]. Values from the three replicate datasets obtained during the experiment are included. The y-axis of the black square box in the Solid graph has been enlarged and is displayed on the right-hand side of the graph. AMK, Amikacin; AZM, Azithromycin; CFIX, Cefixime; CP, Chloramphenicol; CPFX, Ciprofloxacin; CPZ, Cefoperazone; DOXY, Doxycycline; ENX, Enoxacin; NM, Neomycin; TP, Trimethoprim; AR, aspect ratio; Circ, circularity; MaxFeret, maximum Feret’s diameter; MinFeret, minimum Feret’s diameter; Perim, perimeter; Round, roundness; Solid, solidity.

We included the four lines of each resistant strain without dividing them to find common morphological characteristics in the resistant strain and identify differences from the parental strain. Quantitative differences in each morphological feature between the parental and resistant strains were examined by comparing the mean values obtained from the three datasets (Supplementary Figure S3). As a result, the parameters of most features in the resistant strains showed significant differences (*p* < 0.001) from the parental strain in all datasets. The mean values for many features differed slightly among the resistant strains, although the Solid values showed greater similarity. Standard deviations were relatively large for the majority of features, possibly reflecting variations in cell shape and size in the cell population, a different experiment date, and differences between lines. To further evaluate the variations in features between resistant strains, the ratio of change from the parental strain was calculated using median values. Decreases in Area, Perim, Major, and MaxFeret were observed in most resistant strains, while the changes in both Minor and MinFeret were slightly different between the resistant strains (Figure 2). In addition, the majority of the resistant strains showed increases in both Circ and Round, and a decrease in AR, but the AZM-and TP-resistant strains showed an opposite tendency from other resistant strains. For Solid, a small change ratio was seen in all resistant strains. These results suggested that most of the drug-resistant strains tested displayed morphological differences from the parental strain.

### Comparison of cell morphology between parental and antibiotic-resistant strains

Quantitative differences in each morphological feature between the parental and resistant strains were further examined using histogram intersections to consider the variability in cell populations.

**Figure 3:**
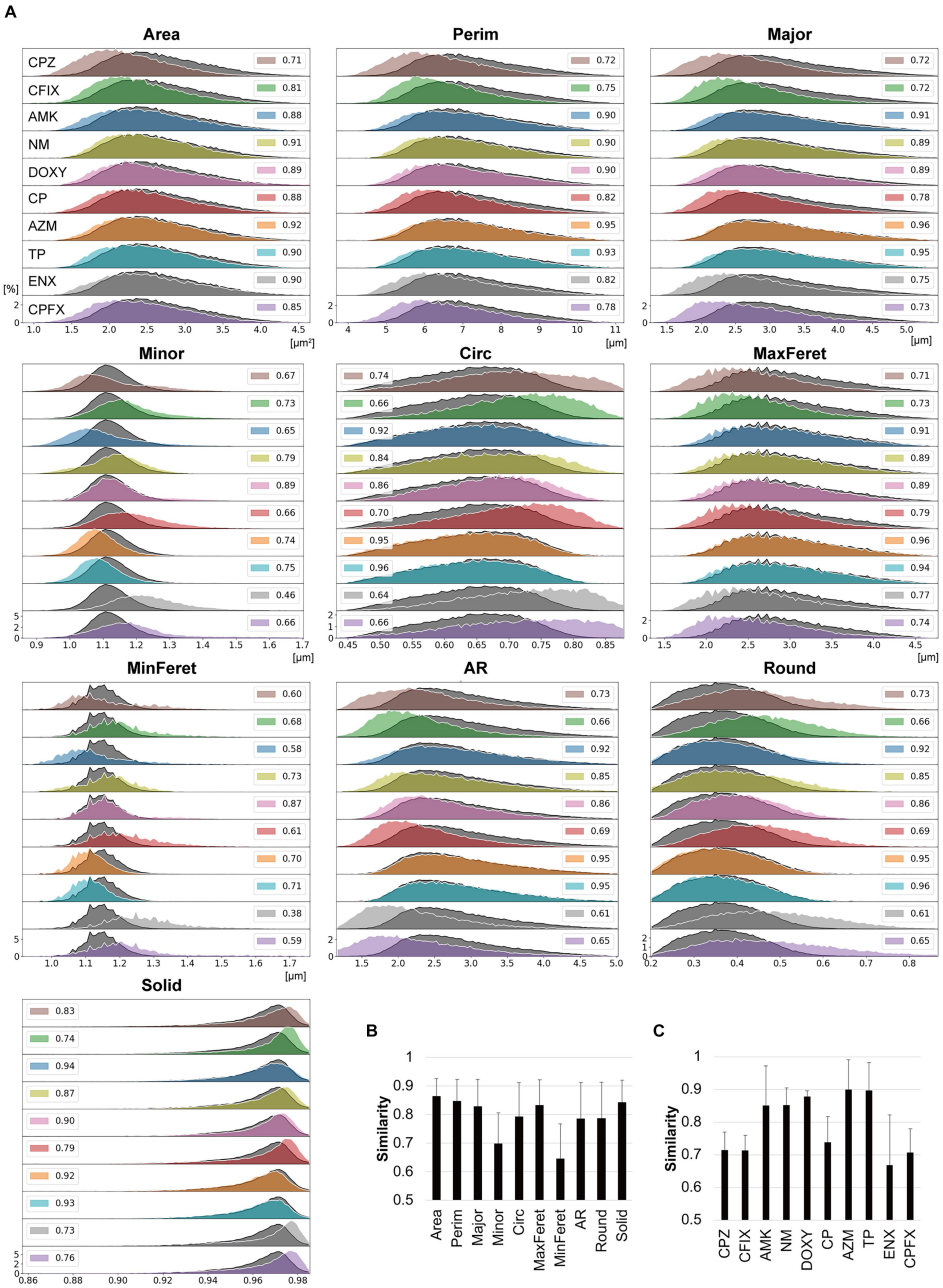
Comparisons between the morphological features of parental and antibiotic-resistant strains using histogram intersections. **(A)** Histograms for each morphological feature measured in the antibiotic-resistant strains are shown in color and outlined in white. The histograms of the parental strain are overlayed in dark gray with black outlines. The numbers provided in the insets indicate the mean similarity values between the parental and resistant strain calculated from the histogram intersections. The horizontal axis indicates the unit value of the measured parameter, and the vertical axis indicates the frequency [%]. **(B,C)** Mean similarity between the parental and antibiotic-resistant strains for each morphological parameter (**B**) and between the parental strain and each antibiotic-resistant strain **(C)** calculated using the histogram intersections. The error bars indicate standard deviation. AMK, Amikacin; AZM, Azithromycin; CFIX, Cefixime; CP, Chloramphenicol; CPFX, Ciprofloxacin; CPZ, Cefoperazone; DOXY, Doxycycline; ENX, Enoxacin; NM, Neomycin; TP, Trimethoprim; AR, aspect ratio; Circ, circularity; MaxFeret, maximum Feret’s diameter; MinFeret, minimum Feret’s diameter; Perim, perimeter; Round, roundness; Solid, solidity.

First, the similarities between three datasets in terms of the morphological parameters in each bacterial strain were examined to evaluate the reproducibility of the data between the datasets. The overall mean similarity was about 0.9, ranging from 0.83 to 0.93 between parameters and from 0.88 to 0.92 between bacterial strains, suggesting that the data variation was quite low between the datasets (Supplementary Table S2). Thus, we included the three datasets in the histogram intersection and asked whether the similarity of each feature between the parental and resistant strains was lower than the similarity between datasets (<0.83). In Figure 3A, histograms displaying the values obtained for each feature of the resistant strains are overlayed with the corresponding histogram for the parental strain. The histograms for Area, Perim, Major, and MaxFeret in the majority of the resistant strains were shifted slightly to the left of the parental strain, suggesting that these features were smaller in the resistant strains. In contrast, the histograms for Solid in the majority of the resistant strains were shifted slightly to the right of the parental strain. Surprisingly, the shapes and distributions of the histograms for Minor and MinFeret differed largely among the resistant strains, with some being broader than those of the parental strain. To a certain extent, these features affected the distributions of the Circ, AR, and Round histograms. While the histogram distributions and shapes differed from those of the parental strain for the majority of the resistant strains, the AZM-and TP-resistant strains showed striking overlaps with the parental strain for most features except Minor and MinFeret.

The histogram intersection was calculated to examine the mean similarity values between the parental and resistant strains (Figure 3A, insets). For some features, these values were < 0.8, with the values for MinFeret and Minor being particularly low (Figure 3B), suggesting that these features were related to the large differences in the shapes of the histograms for the parental and resistant strains (Figure 3A). The CPZ-, CFIX-, CP-, and CPFX-resistant strains, and the ENX-resistant strain in particular, exhibited the lowest similarity to the parental strain (Figure 3C). Conversely, the AZM-and TP-resistant strains showed particularly high similarity to the parental strain.

### Clustering of the antibiotic-resistant strains according to morphological features

The findings described above suggested that the resistant strains had different degrees of morphological similarity to the parental strain. Thus, we considered what morphological tendencies seen in the resistant strains differed from those seen in the parental strain, although the histogram distributions suggested that all resistant strains showed variation in cell shape but many of the cells resembled the parental strain (Figure 3A). Therefore, we attempted to extract morphological characteristics that were shared by the resistant strains. This was done by dividing the cell shapes into different clusters using the k-means clustering method.

First, k-means clustering was conducted for all feature data obtained from the bacterial cells, with the number of clusters set to six. To determine the number of clusters, observations from the microscopy images and the results of the histogram analysis were used to group the bacterial cells into different types with respect to shape and size. A PCA was then applied to each cluster to reveal the characteristics of the clustered cell features. Furthermore, the average shape of the cells in each cluster was visualized by calculating the mean and standard deviation of the contour point coordinates.

**Figure 4:**
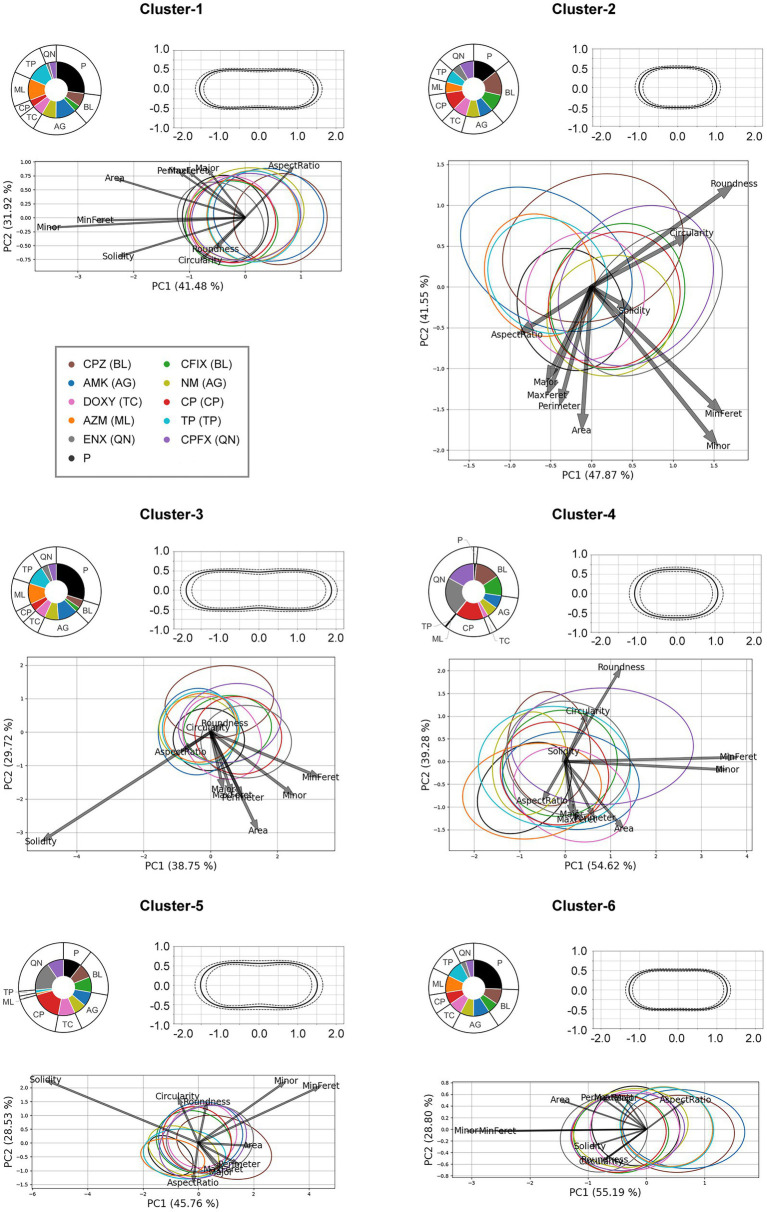
The results of k-means clustering and principal component analysis (PCA) of each cluster. *Upper left panel for each cluster*: The pie charts indicate the proportion of the cluster occupied by each bacterial strain. *Upper right panel for each cluster*: The average shape of the cells in the cluster is shown; the solid line represents the mean and the dotted lines represent the standard deviation. The vertical and horizontal axes are in μm. *Bottom panel for each cluster*: The results of the PCA for each cluster are shown. The ellipse contains approximately 68% of the cells from each strain. The arrows represent the loadings of each feature. P, the parental strain; BL, β-lactam-, AG, aminoglycoside-, TC, tetracycline-, CP, chloramphenicol-, ML, macrolide-, TP, trimethoprim-, QN, quinolone-resistant strains; MaxFeret, maximum Feret’s diameter; MinFeret, minimum Feret’s diameter.

The results of the clustering analysis are presented in Figure 4 and the numbers of cells in each cluster are provided in Supplementary Table S3. The proportion of the bacterial strain in each cluster is visualized using a pie chart. The parental strain was classified in most clusters; however, it was largely excluded from Cluster-4, and surprisingly, the proportions of QN-, BL-, and CP-resistant strains was larger in this cluster. Notably, the proportions of the TP-, ML-, and tetracycline (TC)-resistant strains in Cluster-4 was very small, suggesting that there might be a peculiar morphological characteristic shared by the QN-, BL-, and CP-resistant strains causing them to be clustered together. In addition, clusters containing the most parental strain included less of the QN-, BL-, and CP-resistant strains. However, the presence of both the TP-and ML-resistant strains coincided with that of the parental strain, implying that their morphological characteristics were similar. All clusters contained the aminoglycoside (AG)-resistant strain in similar proportions. The results of the PCA suggested that the Minor and MinFeret features in Cluster-4 were most strongly correlated with the first principal component, while Round was correlated with the second principal component, which was in agreement with the morphological characteristics found in this cluster. The average shapes of the cells differed in each cluster, suggesting that the various cell morphologies of the different bacterial strains were classified into distinct clusters. For example, the average shapes of the cells in Cluster-2, -4, and -6 were rod-like, with different widths or lengths. Cells were fattest in Cluster-4. Cells in Cluster-1, -3, and -5 were elongated and slightly concave around the center. The PCA showed the correlation with Solid was strongest in these three clusters, suggesting that they might contain cells at the onset of division.

The proportions of the six clusters for each bacterial strain were also examined (Supplementary Figure S4). The results revealed large proportions of the parental strain were made up of Cluster-1 and Cluster-6 cells, and a similar tendency was seen in both the TP-and ML-resistant strains. However, the other resistant strains contained smaller proportions of these two clusters. Interestingly, although very small proportions of the parental strain, TP-, and ML-resistant strains were comprised of Cluster-4 and -5, larger proportions of the other resistant strains, particularly the QN-, CP-, and BL-resistant strains were composed of these clusters. The proportion of Cluster-2 cells in most of the resistant strains was slightly larger than in the parental strain.

### Genes correlated with morphological characteristics in the antibiotic-resistant strains

The results of the cluster analysis revealed an increased proportion of fatter or shorter cells in the resistant strains and suggested that they contained a subpopulation of cells with morphological characteristics different to those of the parental strain. Therefore, we investigated the genes associated with the morphological characteristics of the resistant strains.

**Figure 5:**
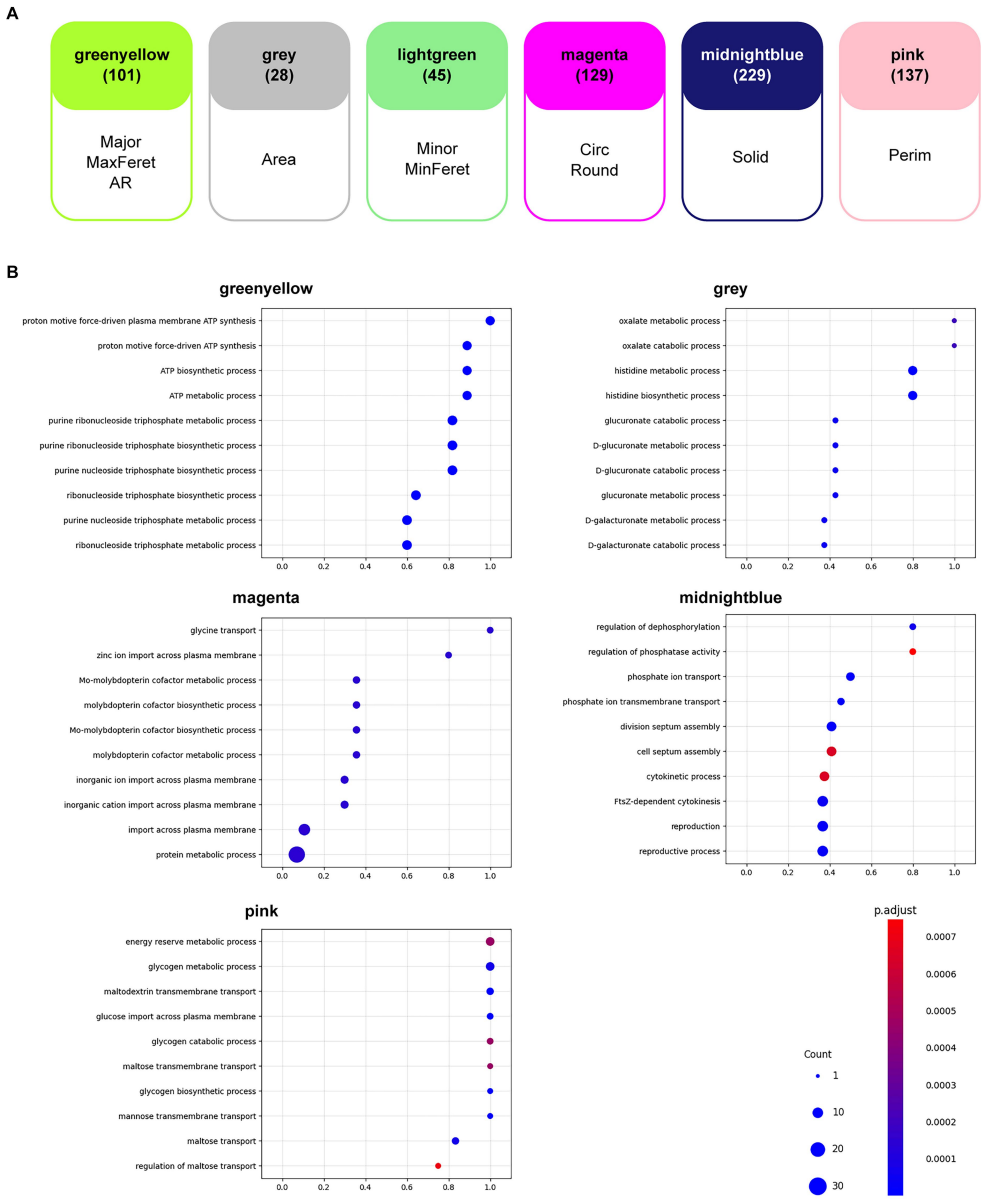
Weighted gene correlation network analysis (WGCNA). **(A)** Gene modules that were most highly correlated with the morphological features analyzed in this study are shown. The number of genes found in each module is shown in parentheses. AR, aspect ratio; Circ, circularity; MaxFeret, maximum Feret’s diameter; MinFeret, minimum Feret’s diameter; Perim, perimeter; Round, roundness; Solid, solidity. **(B)** The results of the gene ontology (GO) enrichment analyses for each of the six gene modules are shown in the bubble charts. The horizontal axis of each chart represents the gene ratio, the size of the bubbles represents the number of genes, and the color of the bubbles represents the false discovery rate (FDR)-adjusted *p*-value.

A WGCNA (Langfelder and Horvath, 2008) was performed to determine the correlation between gene expression (Suzuki et al., 2014) and the morphological features analyzed in this study. As a result, six groups (modules) of genes were found, each of which was most highly correlated with the corresponding morphological feature (Figure 5A). Intermediate results and details of the WGCNA are presented in Supplementary Figure S5 and Supplementary Table S4. A list of genes contained in the six modules is given in Supplementary Table S5.

GO enrichment analysis (Ashburner et al., 2000; Thomas et al., 2022; Aleksander et al., 2023) for the term biological processes in the six modules was performed to determine the tendencies that affected resistant strain morphology. Among the significantly enriched GO terms (FDR < 0.05), the top 10 GO terms with the highest gene ratios are shown in Figure 5B. The observed tendencies in the GO enrichment results suggested that the genes highly correlated with some morphological features were related to cellular energy metabolism. For example, the GO terms in the module “greenyellow,” which was most highly correlated with Major, MaxFeret, and AR, suggested enrichment in energy metabolism, particularly ATP synthesis. Similarly, the GO terms in the module “pink,” which was most highly correlated to Perim, suggested enrichment in cellular processes related to energy reserve metabolism of sugars. Additionally, the GO terms in the module “midnightblue,” which was most highly correlated with Solid, suggested enrichment in the regulation of phosphate metabolism and the cellular events associated with cell division. The GO enrichment results from the other two modules, “gray” and “magenta,” which were most highly correlated to Area and Circ and Round, respectively, suggested enrichment in specialized cellular processes, such as an adaptive metabolic network to efficiently utilize available nutrients and respond to environmental changes, and ion transport and metabolism, which is essential for specific enzymatic functions and important for bacterial survival and adaptation. Although no GO term was enriched in the module “lightgreen,” which was most highly correlated with Min and MinFeret, we focused on this module because it contained genes that are important for antibiotic resistance. In fact, several genes encoding drug efflux pumps or transporters appeared in the module; the *acrA* and *acrB* genes encode proteins that comprise the AcrAB-TolC drug efflux system, known to be a major cause of both intrinsic and acquired resistance to many compounds, including antimicrobials (Okusu et al., 1996), the *mdtG* gene encodes a multidrug efflux pump thought to be involved in resistance to fosfomycin (Nishino and Yamaguchi, 2001), and the *mdlA* and *mdlB* genes encode proteins annotated as putative multidrug resistance-like exporters (Saier et al., 2016). In addition, *acrR*, *soxS*, *soxR*, and *rob*, which are thought to play roles in the regulation of expression of these transporter genes (Ma et al., 1996; Seo et al., 2015; Blanco et al., 2016) appeared in the same module (Table 3).

**Table 3:** List of genes in the module “lightgreen”.^a^

Gene	Description
*yaaY*	DUF2575 domain-containing protein YaaY
*lpxC*	UDP-3-O-acyl-N-acetylglucosamine deacetylase
*dgt*	dGTP triphosphohydrolase
*panE*	2-dehydropantoate 2-reductase
*ybaO*	DNA-binding transcriptional activator DecR
*mdlA*	ABC transporter family protein MdlA
*mdlB*	ABC transporter family protein MdlB
*acrB*	Multidrug efflux pump RND permease AcrB
*acrA*	Multidrug efflux pump membrane fusion lipoprotein AcrA
*acrR*	DNA-binding transcriptional repressor AcrR
*rnk*	Nucleoside diphosphate kinase regulator
*lipA*	Lipoyl synthase
*fur*	DNA-binding transcriptional dual regulator Fur
*fldA*	Flavodoxin 1
*seqA*	Negative modulator of initiation of replication
*ssuA*	Aliphatic sulfonate ABC transporter periplasmic binding protein
*pqiA*	Intermembrane transport protein PqiA
*pqiB*	Intermembrane transport protein PqiB
*ymbA*	Intermembrane transport lipoprotein PqiC
*mdtG*	Efflux pump MdtG
*ribA*	GTP cyclohydrolase 2
*nhoA*	Arylamine N-acetyltransferase
*fumC*	Fumarase C
*zwf*	NADP^+^-dependent glucose-6-phosphate dehydrogenase
*nfo*	Endonuclease IV
*yeiI*	Putative sugar kinase YeiI
*ypeB*	PF12843 family protein YpeB
*ligA*	DNA ligase
*fldB*	Flavodoxin 2
*ygfZ*	Folate-binding protein YgfZ
*yggX*	Putative Fe^2+^-trafficking protein
*mltC*	Membrane-bound lytic murein transglycosylase C
*kdsC*	3-deoxy-D-manno-octulosonate 8-phosphate phosphatase KdsC
*yicM*	Purine ribonucleoside exporter
*yieP*	DNA-binding transcriptional dual regulator YieP
*frvX*	Peptidase M42 family protein FrvX
*sodA*	Superoxide dismutase (Mn)
*kdgT*	2-dehydro-3-deoxy-D-gluconate:H^+^ symporter
*fpr*	Flavodoxin/ferredoxin-NADP^+^ reductase
*soxS*	DNA-binding transcriptional dual regulator SoxS
*soxR*	DNA-binding transcriptional dual regulator SoxR
*ryjA*	Small RNA RyjA
*yjjW*	Putative glycyl-radical enzyme activating enzyme YjjW
*yjjI*	DUF3029 domain-containing protein YjjI
*rob*	DNA-binding transcriptional dual regulator Rob

a Genes appearing in the module “lightgreen,” which was most highly correlated with the parameters Minor and MinFeret are listed in ascending order of the gene annotation number (Blattner et al., 1997).

Overall, these results strongly suggested that the morphological features of the resistant strains were correlated with changes in the expression of genes involved in cellular energy metabolism and multidrug resistance.

### Single-cell classification between the parental and resistant strains

Recently, deep learning approaches have been applied to microscopy images of bacterial cells and cutting edge algorithms have been developed for automatic cell segmentation, tracking, and antimicrobial susceptibility testing (Lugagne et al., 2020; Cutler et al., 2022). However, to the best of our knowledge, no computational methods for identifying drug-resistant bacteria without using drugs have been investigated to date. As the parental and drug-resistant strains presented different cell morphologies even in the absence of drugs, we investigated whether the resistant strains were discernible from the parental strain using deep learning approaches.

**Figure 6:**
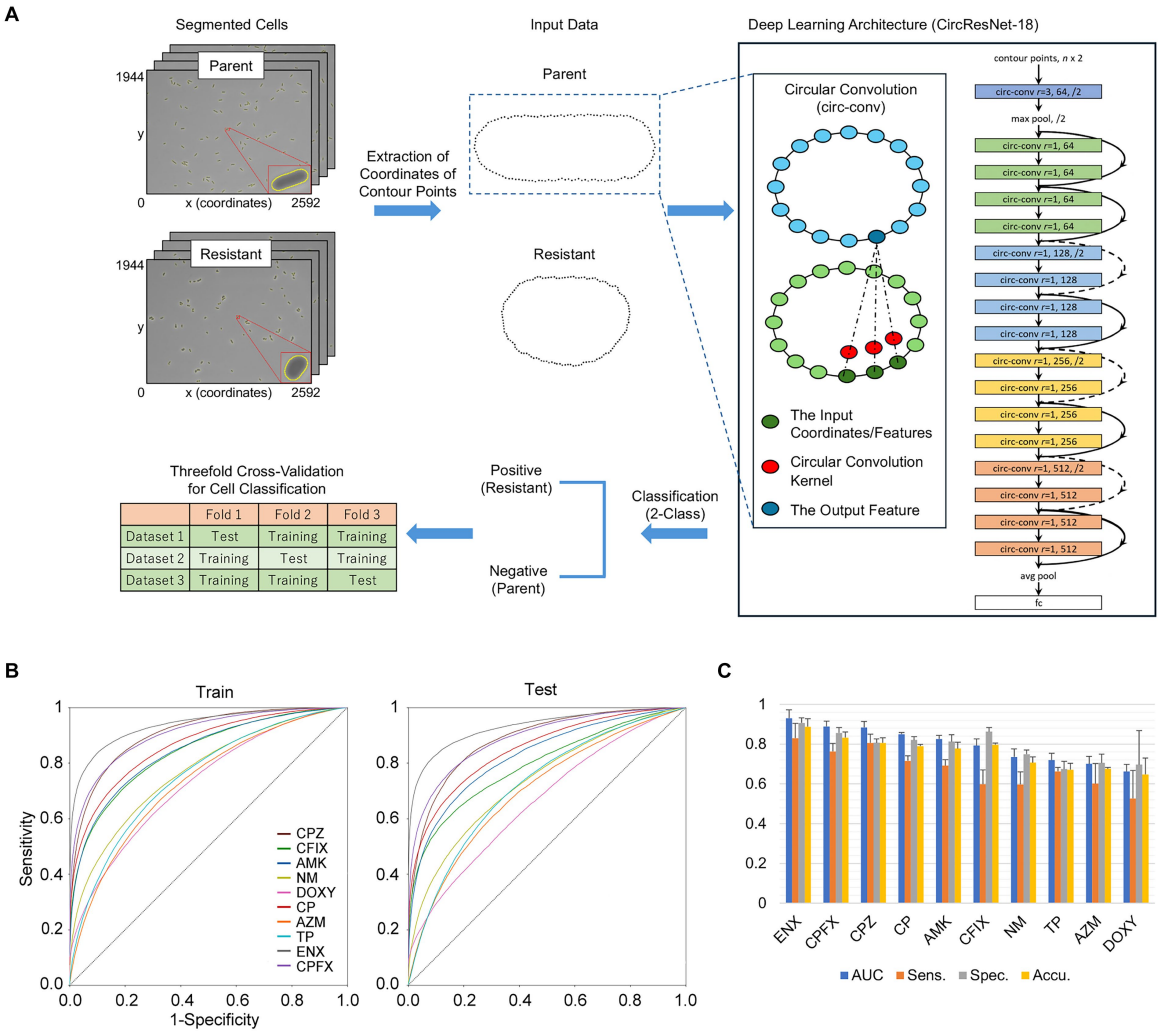
Single-cell classification of parental and antibiotic-resistant cells using deep neural networks. **(A)** The classification workflow. The insets show enlarged views of the contours of the cell region extracted from microscopy images of the parental and resistant (ENX) strains. The corresponding cell contours aligned horizontally and evenly interpolated to 128 points are shown as these formed the input data. A cartoon of the deep learning architecture shows a circular convolution (circ-conv) integrated into the ResNet architecture. For the circ-conv layer, r represents the radius or neighborhood size, resulting in a kernel size of 2r + 1, followed by the number of channels and optionally a stride. The circ-conv layers are followed by batch normalization and rectified linear unit functions. The curved arrows depict a shortcut connection, and the dashed lines indicate the increase in the number of channels. The term fc stands for a fully-connected layer. Threefold cross-validation was conducted for evaluation of the classifier models. **(B)** The receiver operating characteristic (ROC) curve for the contour classification. Each curve shows an average of the ROC curves obtained from the threefold cross-validation. The vertical and horizontal axes represent the values of sensitivity and 1 − specificity, respectively. **(C)** Performance of the network in classifying resistant strains. The classification results (mean values) from the test sets in the threefold cross-validation are presented as bar graphs with standard deviations. The resistant strains are listed in descending order of the area under the curve (AUC). Sens., sensitivity (correctly classified resistant cells); Spec., specificity (correctly classified parental cells). Accu., accuracy (collectly classified parental and resistant cells); AMK, Amikacin; AZM, Azithromycin; CFIX, Cefixime; CP, Chloramphenicol; CPFX, Ciprofloxacin; CPZ, Cefoperazone; DOXY, Doxycycline; ENX, Enoxacin; NM, Neomycin; TP, Trimethoprim; AR, aspect ratio; Circ, circularity; MaxFeret, maximum Feret’s diameter; MinFeret, minimum Feret’s diameter; Perim, perimeter; Round, roundness; Solid, solidity.

We proposed a neural network based on the ResNet architecture and built by replacing all of the two-dimensional convolutions with circular convolutions (Figure 6A). In our preliminary experiments, we employed the original ResNet models for patch classification and found that the classification performance worsened between the training and test phases (Supplementary Figure S6; Supplementary Table S6). These results suggested that the image-based models were substantially affected by the inconsistency in the characteristics seen in the microscopy images between the training and test phases. In the present study, we used cell regions from which the coordinates of contour points were extracted and interpolated as inputs to the ResNet architecture (Figure 6A; Supplementary Table S7) and demonstrated that our contour-based models achieved higher classification performance without suffering from such inconsistency (Figure 6B). As shown by the results of the AUC obtained from the test sets, our classifier models achieved high performance for ENX-, CPFX-, CPZ-, CP-, and AMK-resistant strains (Figure 6C). In addition, the sensitivity, specificity, and accuracy results, defined as Equations 1–3 respectively (see Materials and Methods), showed that the classification performance of our models was higher for specificity than sensitivity in all resistant strains, suggesting that there were fewer false positives than false negatives (Figure 6C). Our classification models showed high performance for sensitivity in the ENX-and CPZ-resistant strains. One of the reasons for the lower performance for sensitivity compared with specificity was that many resistant cells were of a similar shape to the parental cells, which may have made it more difficult for classifier models to learn the differences in shape between resistant and parental cells, therefore increasing the false negatives in the test phases.

## Discussion

Previous studies on bacterial drug resistance have generally focused on changes in the resistance potential caused by specific gene mutations in drug-resistant cells. Similarly, morphological changes associated with particular genes have been described (Hirota et al., 1968; Wachi et al., 1987). In recent years, the analysis of transcriptional data has revealed that the expression patterns of many genes are altered in laboratory-evolved antibiotic-resistant cells, and it is becoming possible to predict drug-resistant bacteria from gene expression profiles (Suzuki et al., 2014; Maeda et al., 2020). Because the expression patterns of multiple genes are altered in antibiotic-resistant strains, it is plausible that these changes may have a complex effect on the morphology of bacterial cells. Studies on the relationship between antibiotic resistance and bacterial morphology have included observations of bacterial cells treated with drugs, and a recent study suggested that changes in bacterial cell shape are important for adaptation to antibiotics and drug resistance (Ojkic et al., 2022). However, the morphology of resistant bacteria in the absence of antibiotics is not well known.

In this study, we showed that the antibiotic-resistant *E. coli* strains that evolved in the laboratory maintained their morphological changes even in the absence of drugs. The strains that evolved more prominent changes in their cell morphology showed higher resistance to the corresponding drug (Supplementary Figure S1), but the relationship between the degree of morphological change and the minimum inhibitory concentration values require further investigation.

Previous reports have indicated that there is a relationship between cells with smaller morphological features, such as persister cells, and drug tolerance (Shah et al., 2006; Lewis, 2010). A relationship between the morphological characteristics of the drug-resistant *E. coli* strains and the genes involved in cellular energy metabolism, cell division, and antibiotic resistance is suggested. The drug-resistant *E. coli* strains have a slower growth rate than the parental strain under antibiotic-free conditions (Suzuki et al., 2014), and this growth characteristic was observed in the present study (Supplementary Figure S7). We observed a similar morphology in resistant strains across different antibiotic mechanisms (QN, BL, CP), suggesting a potential common bacterial survival response. We identified genes highly correlated with morphological features that exhibited significant expression changes in drug-resistant *E. coli* strains (Supplementary Table S8). Notably, *ompF* was present in strains resistant to QN, BL, and CP. Given that *ompF* significantly contributes to drug resistance in laboratory-evolved *E. coli* (Suzuki et al., 2014; Maeda et al., 2020), it is possible that this gene is also associated with both cell morphology and survival response observed in strains resistant to these antibiotics. Considering that some of the genes that regulate persister formation also appeared in the group of genes that were correlated with some of the morphological features measured in this study (Supplementary Table S5), it is possible that changes in the expression of genes that play a role in cellular functions such as those described in this study may have a complex effect on the morphology of drug-resistant strains under long-term antibiotic stress, and this morphology may be maintained in subpopulations even after removal of the stress. The resistant strains used in the present study were obtained through long-term exposure to the corresponding antibiotics, and when the morphological changes took place is currently unknown; moreover, the mechanism by which subpopulations with revised cell morphologies are generated from single colonies of drug-resistant strains is unclear. They may be generated through uneven division of cells that are similar in shape to the parental strain, or possibly cells with distinct morphology are preferable and therefore maintained as the subpopulation.

It should be noted, however, that the interpretation of the results is limited by the sole use of experimentally-evolved strains and morphological information obtained from light microscopy images; the internal structures of most of these resistant cells have yet to be studied. Future investigations are required to clarify whether the acquisition of drug resistance is generally associated with changes in cell morphology and what genes are responsible, using various drug-resistant bacterial strains obtained under other environmental conditions such as clinical isolates.

Finally, our proposed single-cell classification demonstrated a high level of performance in characterizing some of the drug-resistant strains. The classification results generally coincided with the findings from the morphological analysis in that the accuracy in discrimination between the parental and resistant strains reflected both the similarities in cell shape and the minimum inhibitory concentration values. A future challenge will be to develop an algorithm that enables the classification of cells in a heterogenous population.

## Data availability statement

The datasets presented in this study can be found in the online repository; https://doi.org/10.6084/m9.figshare.c.7757147.v1.

## Author contributions

MI: Formal analysis, Investigation, Methodology, Writing – original draft. KA: Formal analysis, Investigation, Methodology, Writing – original draft. MH-N: Conceptualization, Formal analysis, Funding acquisition, Investigation, Methodology, Supervision, Writing – original draft. CF: Conceptualization, Resources, Writing – review & editing. KN: Funding acquisition, Project administration, Supervision, Writing – review & editing.

## Footnotes

^1^ https://github.com/kevinjohncutler/omnipose

^2^ http://www.pantherdb.org
