# Erratum: Bioinformatic analysis reveals the association between bacterial morphology and antibiotic resistance using light microscopy with deep learning

**DOI:** 10.3389/fmicb.2024.1516320

**Published:** 2024-11-08

**Authors:** Frontiers Production Office

**Affiliations:** Frontiers Media SA, Lausanne, Switzerland

**Keywords:** antibiotic resistance, light microscopy, bacterial morphology, deep learning, bioinformatic analysis

Due to a production error, the link to the supplementary material for this article led to incorrect files.

The publisher apologizes for this mistake.

The original version of this article has been updated.

