# Corrigendum: Bioinformatic analysis reveals the association between bacterial morphology and antibiotic resistance using light microscopy with deep learning

**DOI:** 10.3389/fmicb.2025.1607769

**Published:** 2025-04-17

**Authors:** Miki Ikebe, Kota Aoki, Mitsuko Hayashi-Nishino, Chikara Furusawa, Kunihiko Nishino

**Affiliations:** ^1^SANKEN (Institute of Scientific and Industrial Research), Osaka University, Osaka, Japan; ^2^Graduate School of Pharmaceutical Sciences, Osaka University, Suita, Japan; ^3^Artificial Intelligence Research Center (AIRC-SANKEN), Osaka University, Osaka, Japan; ^4^Center for Biosystems Dynamics Research, RIKEN, Suita, Japan; ^5^Universal Biology Institute, The University of Tokyo, Tokyo, Japan; ^6^Center for Infectious Disease Education and Research, Osaka University, Osaka, Japan

**Keywords:** antibiotic resistance, light microscopy, bacterial morphology, deep learning, bioinformatic analysis

In the published article, there was an error in the **Data availability statement**. It was incorrectly stated that the names of the repository (and accession number) can be found in the article or Supplementary material. The correct Data Availability statement appears below.

## Data Availability

The datasets presented in this study can be found in the online repository; https://doi.org/10.6084/m9.figshare.c.7757147.v1. The authors apologize for this error and state that this does not change the scientific conclusions of the article in any way. The original article has been updated.

