# Supplementary material for: Bioinformatic analysis reveals the association between bacterial morphology and antibiotic resistance using light microscopy with deep learning
Source: Front Microbiol. 2024 Sep 19;15:1450804. doi: 10.3389/fmicb.2024.1450804 (PMC11446759; doi:10.3389/fmicb.2024.1450804)
Supplement: Supplementary file 1 [file Data_Sheet_1.PDF]

SUPPLEMENTARY INFORMATION

Bioinformatic Analysis Reveals the Association between Bacterial Morphology and Antibiotic Resistance using Light Microscopy with Deep Learning

Miki Ikebe, Kota Aoki, Mitsuko Hayashi-Nishino, Chikara Furusawa, Kunihiko Nishino

Supplementary Materials

Supplementary Methods for Preparation of M9 Medium

Fig. S1. Minimum inhibitory concentrations of antibiotics required to restrict the growth of the antibiotic-resistant strains of *Escherichia coli* used in the study.
Fig. S2. Examples of cell segmentation using Omnipose.
Fig. S3. Comparisons between the parameters of the morphological features measured in the parental and antibiotic-resistant strains of *Escherichia coli*.
Fig. S4. The proportions of six clusters of morphological features in each strain of antibiotic-resistant *Escherichia coli*.
Fig. S5. Weighted gene correlation network analysis (WGCNA).
Fig. S6. The results of patch classification of the parental and antibiotic-resistant strains of *Escherichia coli*.
Fig. S7. Growth curves of the parental and antibiotic-resistant strains of *Escherichia coli*.
Table S1. Numbers of cells used in the study.
Table S2. Similarity between three sets of data on the morphological features measured in antibiotic-resistant strains of *Escherichia coli*.
Table S3. The results of k-means clustering.
Table S4. Number of genes in each of the 19 modules obtained using a weighted gene correlation network analysis.
Table S5. Lists of genes in each of six gene modules highly correlated with the morphological features measured in this study.
Table S6. Number of patches used for patch classification.
Table S7. Number of cells used for single-cell classification.

Table S8. Genes significantly different in their expression levels between each resistant strain and the parental strain and highly correlated with morphological features.

Supplementary Methods for Preparation of M9 Medium

Preparation of 1x M9 Liquid Culture Medium (1L)

1. **Ingredients:**

   - Glucose: 5.0 g
   - $Na_2HPO_4 \cdot 12H_2O$: 17.1 g
   - KH_2PO_4: 3.0 g
   - NH_4Cl: 2.0 g
   - NaCl: 0.5 g

2. **Stock Solutions (1000x):**

   - 1000x $CaCl_2 \cdot 2H_2O$: 1 mL
   - 1000x $MgSO_4 \cdot 7H_2O$: 1 mL
   - 1000x $FeSO_4 \cdot 7H_2O$: 1 mL
   - 1000x Thiamine·HCl: 1 mL

3. **Procedure:**

   1. Dissolve the ingredients in approximately 900 mL of Milli-Q water.
   2. Adjust pH to 7.0 with phosphoric acid (H_3PO_4).
   3. Adjust the final volume to 1 liter with Milli-Q water.
   4. Sterilize the medium by filtering through a 0.22 μm filter.
   5. Store the medium at 4°C with protection from light.

Supplementary Methods for Preparation of M9 Medium (Continued)

Preparation of 1000x Stock Solutions

1. **$CaCl_2 \cdot 2H_2O$**

   - $CaCl_2 \cdot 2H_2O$: 2.94 g
   - Adjust to 200 mL with MilliQ water.
   - Divide into 1 mL aliquots.

2. **$MgSO_4 \cdot 7H_2O$**

   - $MgSO_4 \cdot 7H_2O$: 24.6 g
   - Adjust to 200 mL with MilliQ water.
   - Divide into 1 mL aliquots.

3. **FeSO4·7H2O**

   - $FeSO_4 \cdot 7H_2O$: 556 mg
   - Adjust to 200 mL with MilliQ water.
   - Divide into 1 mL aliquots.

4. **Thiamine·HCl**

   - Thiamine·HCl: 2.0 g
   - Adjust to 200 mL with MilliQ water.
   - Divide into 1 mL aliquots in opaque container.

Store 1 mL aliquots at -20°C.

Supplementary Methods for Preparation of M9 Medium (Continued)

Preparation of 2x M9 Agar Medium (1L)

1. **Ingredients:**

   - Glucose: 10 g
   - $Na_2HPO_4 \cdot 12H_2O$: 34.2 g
   - KH_2PO_4: 6.0 g
   - NH_4Cl: 4.0 g
   - NaCl: 1.0 g

2. **Stock Solutions (1000x):**

   - 1000x $CaCl_2 \cdot 2H_2O$: 2 mL
   - 1000x $MgSO_4 \cdot 7H_2O$: 2 mL
   - 1000x $FeSO_4 \cdot 7H_2O$: 2 mL
   - 1000x Thiamine·HCl: 2 mL

3. **Procedure:**

   1. Dissolve the ingredients in approximately 900 mL of Milli-Q water.
   2. Adjust pH to 7.0 with phosphoric acid (H_3PO_4).
   3. Adjust the final volume to 1 liter with Milli-Q water.
   4. Sterilize the medium by filtering through a 0.22 µm filter.
   5. Store the medium at 4°C with protection from light.

Supplementary Methods for Preparation of M9 Medium (Continued)

Preparation of 1x M9 Agar Plates (1.5% Agar)

1. Prepare 2x M9 medium:

    - Follow the recipe for 2x M9 medium preparation.
    - Heat to approximately 60°C.

2. Prepare 3% agar solution:

    - Dissolve 3 g of agar in 100 mL of water.
    - Autoclave the agar solution.

3. Cool the autoclaved agar solution to around 80°C.
4. Mix equal volumes of the 2x M9 medium (warmed to 60°C) and the autoclaved 3% agar solution.
5. Pour the mixture into Petri dishes, ensuring a thick layer.

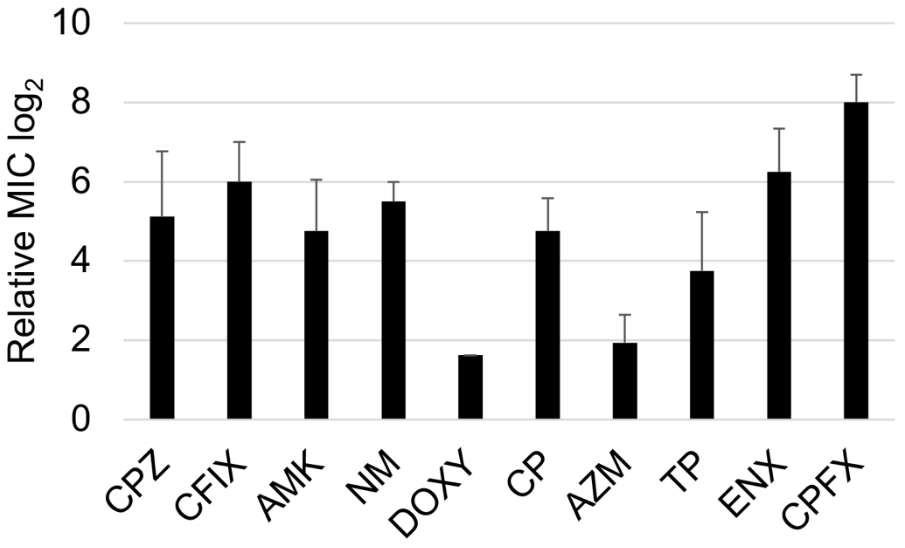

Fig. S1. Minimum inhibitory concentrations of antibiotics required to restrict the growth of the antibiotic-resistant strains of *Escherichia coli* used in the study.
The $\log_2$ transformed average minimum inhibitory concentration (MIC) of each antibiotic for single colony isolates of antibiotic-resistant strains relative to the parental strain. Four lines of each resistant strain were tested. Error bars represent the standard deviation. The difference in the relative MIC $\log_2$ values between the isolates of the resistant strains and the original resistant strains from Suzuki et al. (2014) calculated in this study was not significant ($p < 0.05$) as determined by a Mann-Whitney U test.
AMK = Amikacin; AZM = Azithromycin; CFIX = Cefixime; CP = Chloramphenicol; CPFX = Ciprofloxacin; CPZ = Cefoperazone; DOXY = Doxycycline; ENX = Enoxacin; NM = Neomycin; TP = Trimethoprim.

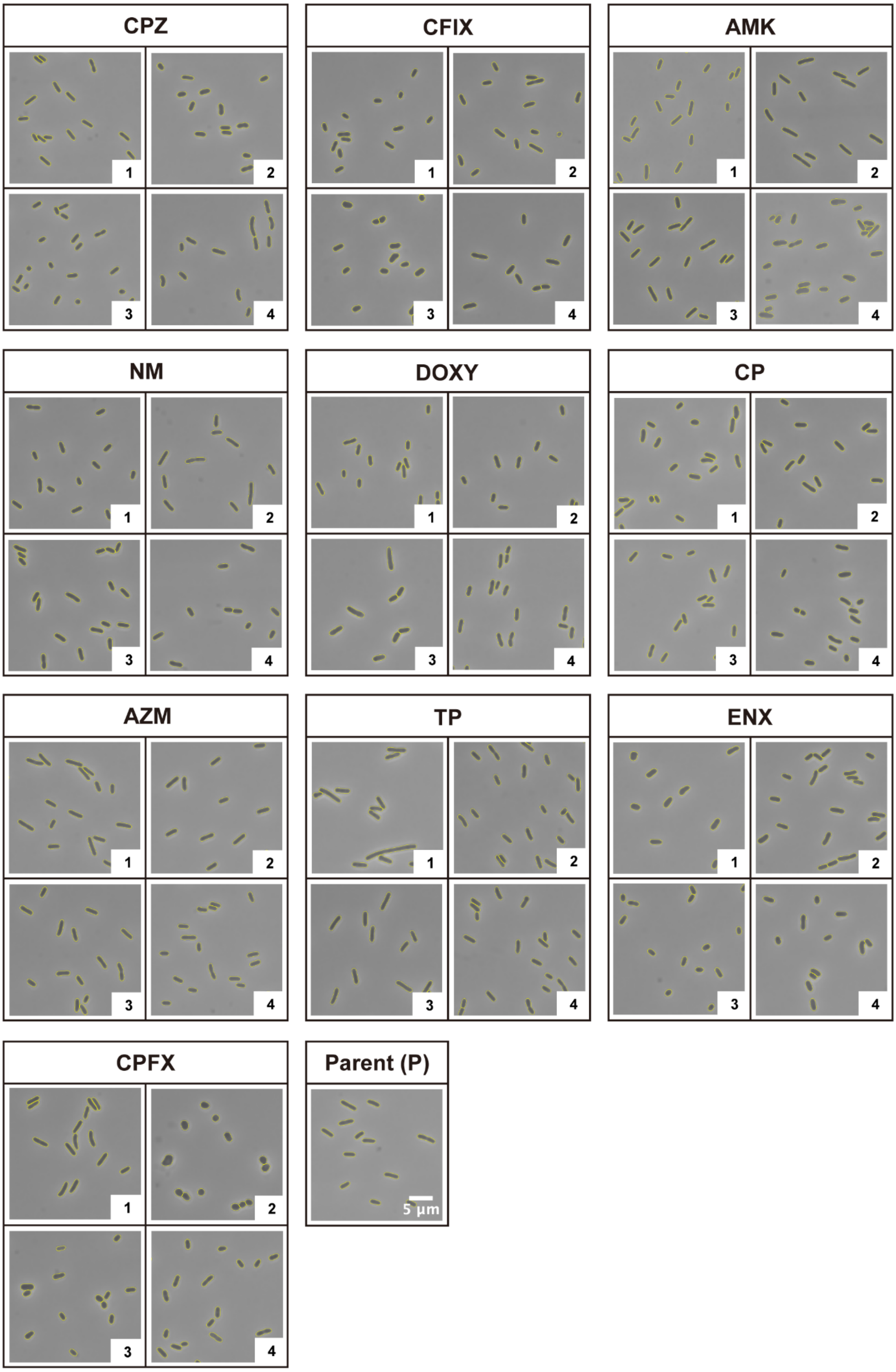
CPZ
1
2
3
4
CFIX
1
2
3
4
AMK
1
2
3
4
NM
1
2
3
4
DOXY
1
2
3
4
CP
1
2
3
4
AZM
1
2
3
4
TP
1
2
3
4
ENX
1
2
3
4
CPFX
1
2
3
4
Parent (P)
5 µm

Fig. S2. Examples of cell segmentation using Omnipose.

The outlines of bacterial cell regions detected using Omnipose were extracted (shown in yellow lines) and overlayed on the original light microscopy images of the antibiotic-resistant and parental strains. Examples of segmentation results are presented from one of three dataset (Dataset 1) in 600 × 600-pixel images cropped from 1,944 × 2,592-pixel images. Four lines of each resistant strain were tested and the inset numbers indicate the line shown. The scale bar shown in the image of the parental strain applies to all panels. AMK = Amikacin; AZM = Azithromycin; CFIX = Cefixime; CP = Chloramphenicol; CPFX = Ciprofloxacin; CPZ = Cefoperazone; DOXY = Doxycycline; ENX = Enoxacin; NM = Neomycin; TP = Trimethoprim.

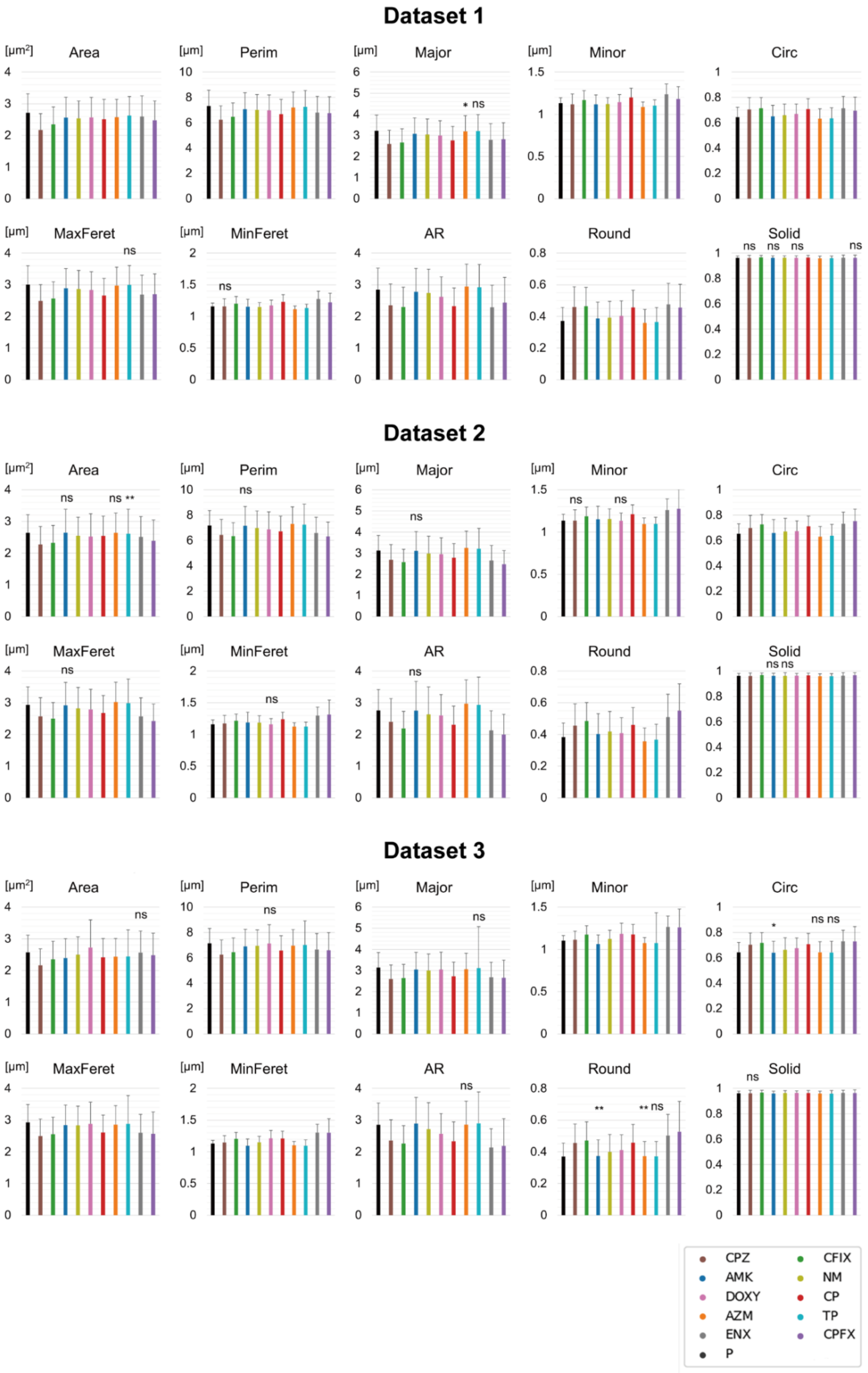
Dataset 1
Area
Perim
Major
Minor
Circ
MaxFeret
MinFeret
AR
Round
Solid
Dataset 2
Area
Perim
Major
Minor
Circ
MaxFeret
MinFeret
AR
Round
Solid
Dataset 3
Area
Perim
Major
Minor
Circ
MaxFeret
MinFeret
AR
Round
Solid
[µm²]
[µm]
CPZ
CFIX
AMK
NM
DOXY
CP
AZM
TP
ENX
CPFX
P

Fig. S3. Comparisons between the parameters of the morphological features measured in the parental and antibiotic-resistant strains of *Escherichia coli.*

Bar graphs show the measurements (mean values) of the morphological features in each bacterial strain obtained from three datasets. The error bars represent standard deviations. All values were significantly different from the parental strain ($P < 0.001$, Welch's t test), unless indicated by * ($P < 0.05$), ** ($P < 0.01$), or ns (no significant difference). AMK = Amikacin; AZM = Azithromycin; CFIX = Cefixime; CP = Chloramphenicol; CPFX = Ciprofloxacin; CPZ = Cefoperazone; DOXY = Doxycycline; ENX = Enoxacin; NM = Neomycin; TP = Trimethoprim; AR = aspect ratio; Circ = circularity; MaxFeret = maximum Feret's diameter; MinFeret = minimum Feret's diameter; Perim = perimeter; Round = roundness; Solid = solidity.

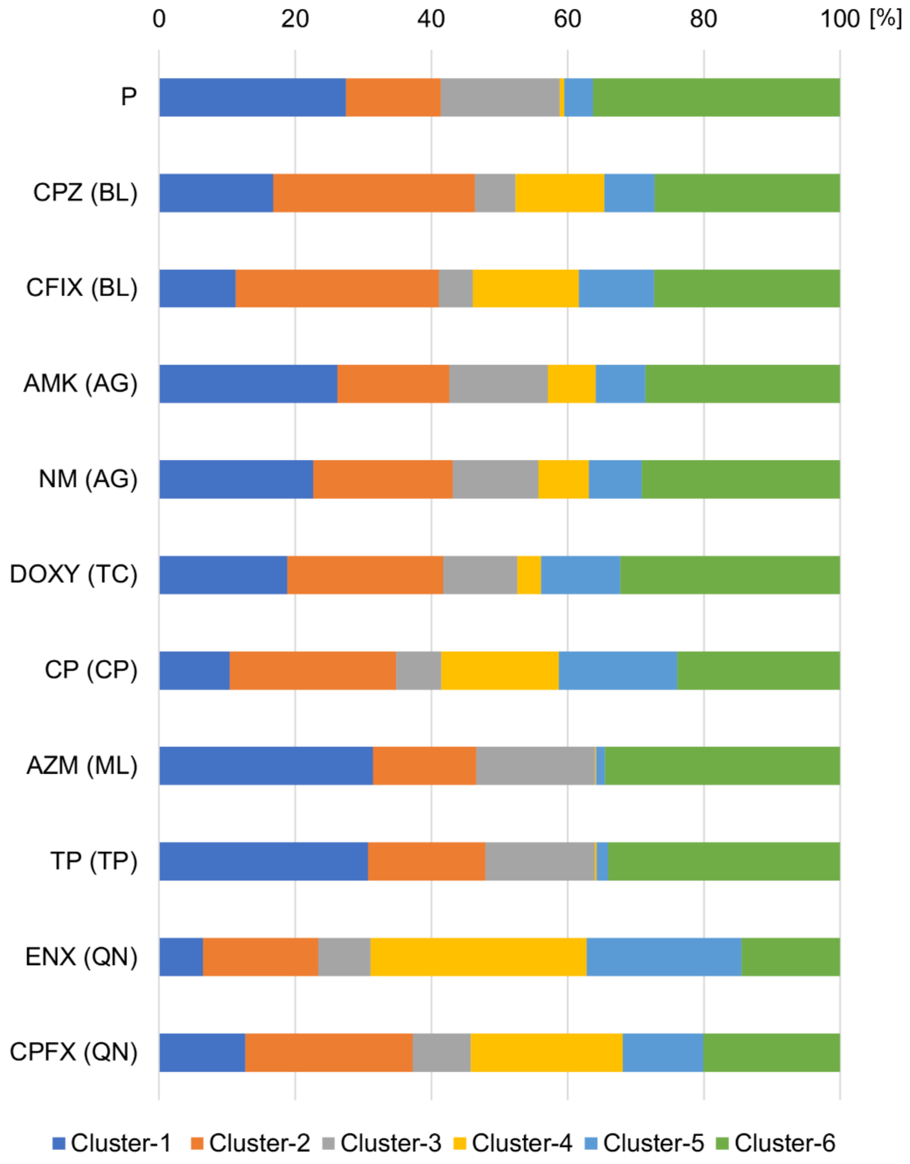

Fig. S4. The proportions of six clusters of morphological features in each strain of antibiotic-resistant *Escherichia coli*.

The proportions of the cell population from each antibiotic-resistant strain represented by six clusters of morphological features. AMK = Amikacin; AZM = Azithromycin; CFIX = Cefixime; CP = Chloramphenicol; CPFX = Ciprofloxacin; CPZ = Cefoperazone; DOXY = Doxycycline; ENX = Enoxacin; NM = Neomycin; TP = Trimethoprim.

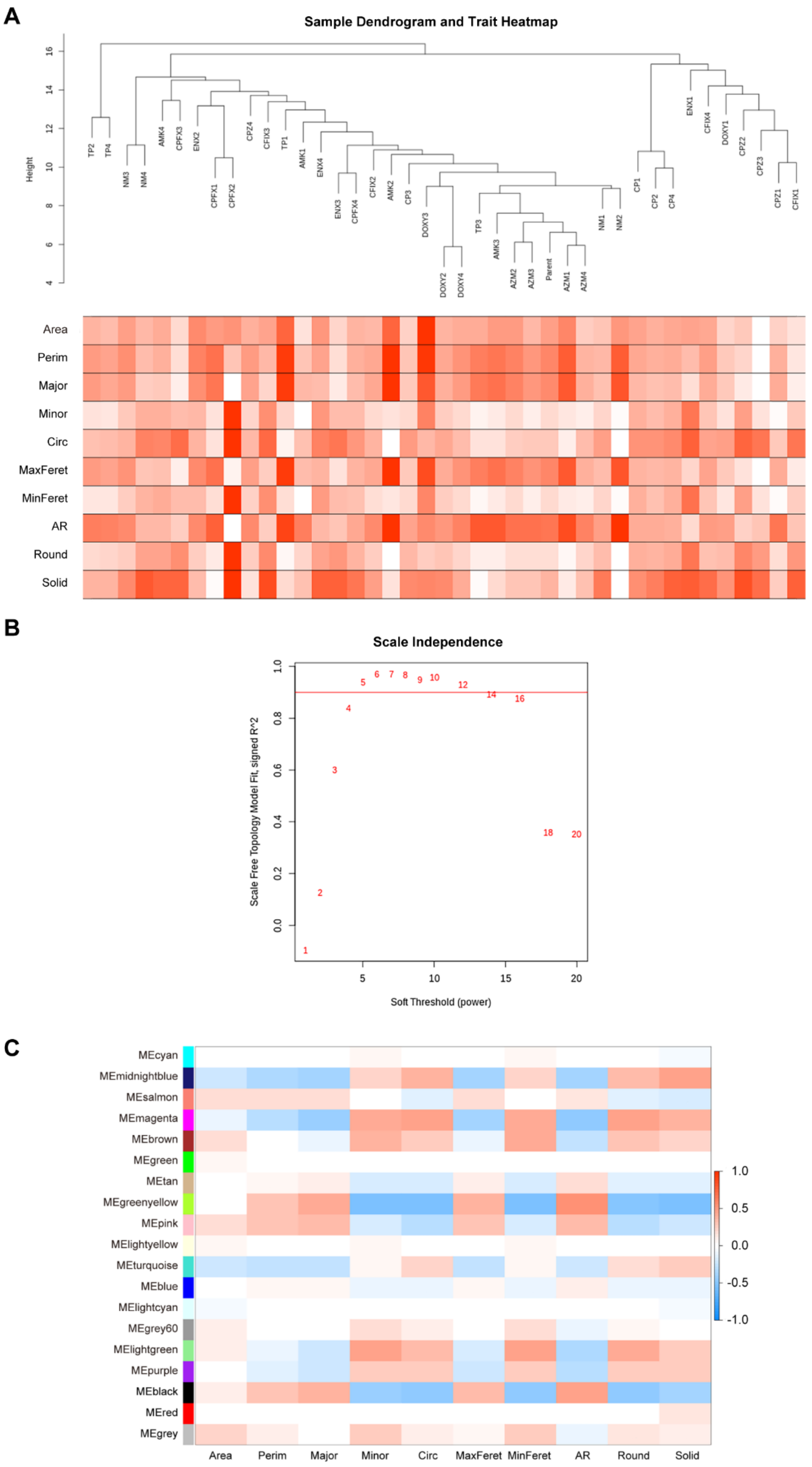
A
Sample Dendrogram and Trait Heatmap
Height
TP2 TP4 NM3 NM4 AMK4 CPFX3 ENX2 CPFX1 CPFX2 CPZ4 CFIX3 TP1 AMK1 ENX4 ENX3 CPFX4 CFIX2 AMK2 CP3 DOXY3 DOXY2 DOXY4 TP3 AMK3 AZM2 AZM3 Parent AZM1 AZM4 NM1 NM2 CP1 CP2 CP4 ENX1 CFIX4 DOXY1 CPZ2 CPZ3 CPZ1 CFIX1
Area
Perim
Major
Minor
Circ
MaxFeret
MinFeret
AR
Round
Solid
B
Scale Independence
Scale Free Topology Model Fit, signed R^2
Soft Threshold (power)
C
MEcyan
MEmidnightblue
MEsalmon
MEmagenta
MEbrown
MEgreen
MEtan
MEgreenyellow
MEpink
MElightyellow
MEturquoise
MEblue
MElightcyan
MEgrey60
MElightgreen
MEpurple
MEblack
MEred
MEgrey
Area Perim Major Minor Circ MaxFeret MinFeret AR Round Solid
1.0 0.5 0.0 -0.5 -1.0

Fig. S5. Weighted gene correlation network analysis (WGCNA).

A: Hierarchical clustering performed for the parental strain and 40 antibiotic-resistant strains of *Escherichia coli* based on the Euclidean distance of the gene expression levels are shown as the sample dendrogram with traits heatmap. In the trait heatmap, the median value of each feature is visualized. Darker red color indicates larger values.

B: Analysis of network topology for various soft threshold powers. The x-axis represents the soft threshold power, and the y-axis represents the scale-free fit index. Soft threshold powers were tested from 1 to 20, and 5 was selected, with which the scale-free R^2 reached 0.9 (indicated by the red line).

C: Module-trait associations. Each row corresponds to a module, and each column to a trait (i.e., a quantitative feature). The table is color-coded by correlation according to the color bar.

AR = aspect ratio; Circ = circularity; MaxFeret = maximum Feret's diameter; MinFeret = minimum Feret's diameter; Perim = perimeter; Round = roundness; Solid = solidity.

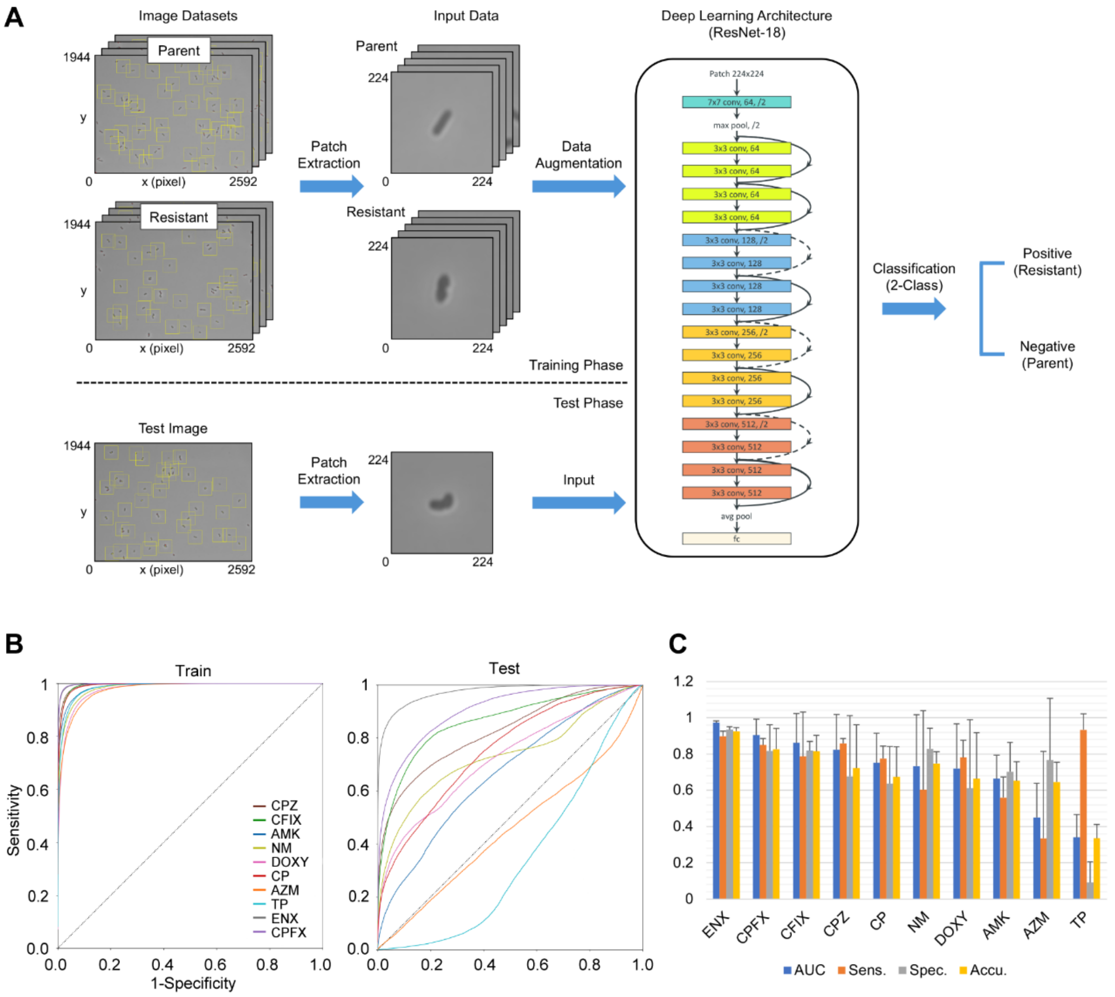

Fig. S6. The results of patch classification of the parental and antibiotic-resistant strains of *Escherichia coli*.

A: A diagram of the patch classification of the parental and resistant strains. Yellow squares in the microscopy images indicate the 224 × 224 patches used for input data. A cartoon of the ResNet model for patch classification is shown.

B: Receiver Operating Characteristic (ROC) curve for the patch classification. The vertical and horizontal axes represent the values of sensitivity and 1 − specificity, respectively. Each curve shows an average of the ROC curves obtained in the threefold cross-validation.

C: Classification performance for the antibiotic-resistant strains. Mean values of the classification results of the test sets in the threefold cross-validation are presented as bar graphs with standard deviations. The resistant strains are listed in descending order of the area under the curve (Gene Ontology et al.). Sens. indicates sensitivity (correctly classified resistant cells), and Spec. indicates specificity (correctly classified parental cells). Accu. indicates accuracy (collectly classified parental and resistant cells). AMK

= Amikacin; AZM = Azithromycin; CFIX = Cefixime; CP = Chloramphenicol; CPFX = Ciprofloxacin; CPZ = Cefoperazone; DOXY = Doxycycline; ENX = Enoxacin; NM = Neomycin; TP = Trimethoprim.

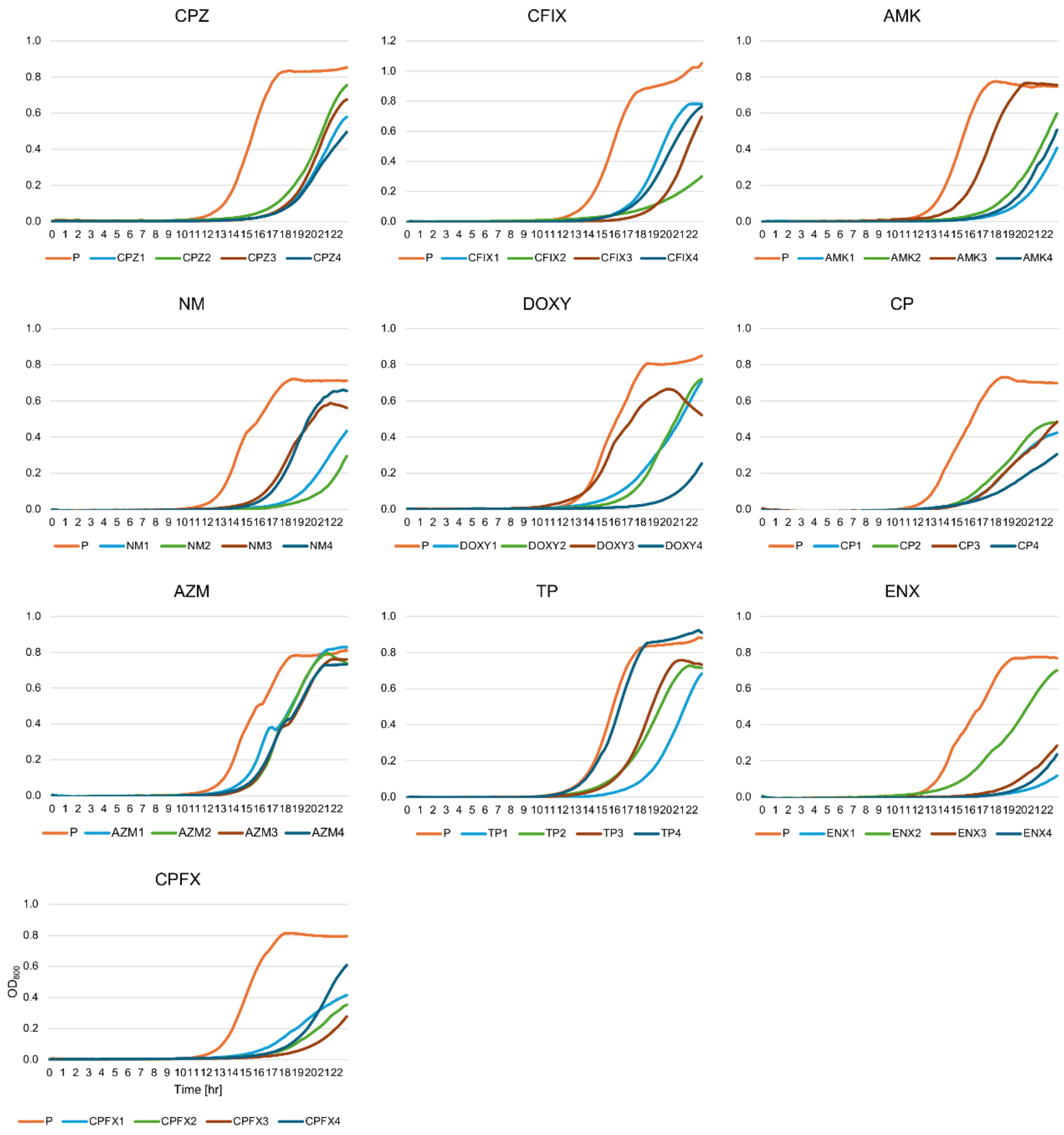

Fig. S7. Growth curves of the parental and antibiotic-resistant strains of *Escherichia coli*.

The growth curves of the parental and antibiotic-resistant strains are shown. All curves represent data from a 23-hour incubation at 34°C in the preculture of Dataset 1. The vertical and horizontal axes represent the values of optical density $(OD)_{600\ nm}$ and the incubation periods (hours), respectively. AMK = Amikacin; AZM = Azithromycin; CFIX = Cefixime; CP = Chloramphenicol; CPFX = Ciprofloxacin; CPZ = Cefoperazone; DOXY = Doxycycline; ENX = Enoxacin; NM = Neomycin; P = Parental strain; TP = Trimethoprim.

Table S1. Numbers of cells used in the study[a].

	Dataset 1	Dataset 2	Dataset 3	Total
**P**	18,937	20,765	19,664	59,366
**CPZ**	7,728	13,063	6,848	27,639
**CFIX**	6,502	5,874	6,839	19,215
**AMK**	11,331	5,845	10,250	27,426
**NM**	6,062	10,775	5,439	22,276
**DOXY**	6,670	5,440	8,052	20,162
**CP**	9,015	8,786	6,665	24,466
**AZM**	8,033	6,953	9,004	23,990
**TP**	8,507	6,581	9,583	24,671
**ENX**	7,040	6,608	4,186	17,834
**CPFX**	6,856	5,808	6,784	19,448

[a]The numbers of cells used for the analyses. Datasets 1–3 are datasets collected from cells cultured on different dates.

AMK = Amikacin; AZM = Azithromycin; CFIX = Cefixime; CP = Chloramphenicol; CPFX = Ciprofloxacin; CPZ = Cefoperazone; DOXY = Doxycycline; ENX = Enoxacin; NM = Neomycin; TP = Trimethoprim.

Table S2. Similarity between three sets of data on the morphological features measured in antibiotic-resistant strains of *Escherichia coli*[a].

	Area	AR	Circ	Major	Max Feret	Min Feret	Minor	Perim	Round	Solid	mean
**P**	0.93	0.95	0.95	0.95	0.94	0.81	0.84	0.94	0.95	0.94	0.92
**CPZ**	0.93	0.93	0.93	0.93	0.92	0.87	0.87	0.94	0.93	0.95	0.92
**CFIX**	0.92	0.91	0.91	0.93	0.92	0.81	0.83	0.93	0.91	0.93	0.90
**AMK**	0.89	0.90	0.90	0.93	0.91	0.76	0.77	0.92	0.91	0.89	0.88
**NM**	0.93	0.92	0.92	0.93	0.92	0.84	0.86	0.93	0.92	0.93	0.91
**DOXY**	0.92	0.93	0.93	0.92	0.92	0.89	0.89	0.92	0.93	0.92	0.92
**CP**	0.92	0.95	0.94	0.93	0.92	0.85	0.86	0.94	0.94	0.94	0.92
**AZM**	0.91	0.93	0.93	0.91	0.91	0.85	0.87	0.91	0.94	0.94	0.91
**TP**	0.88	0.93	0.92	0.91	0.89	0.75	0.80	0.89	0.93	0.93	0.88
**ENX**	0.92	0.91	0.91	0.92	0.91	0.88	0.90	0.92	0.91	0.93	0.91
**CPFX**	0.90	0.86	0.87	0.89	0.87	0.84	0.85	0.88	0.86	0.92	0.87
**mean**	0.91	0.92	0.92	0.92	0.91	0.83	0.85	0.92	0.92	0.93	0.90

[a]Histogram intersections were calculated and averaged as the similarity of a feature between each pair of three datasets for each bacterial strain.

AMK = Amikacin; AZM = Azithromycin; CFIX = Cefixime; CP = Chloramphenicol; CPFX = Ciprofloxacin; CPZ = Cefoperazone; DOXY = Doxycycline; ENX = Enoxacin; NM = Neomycin; P = Parental strain; TP = Trimethoprim. AR = aspect ratio; Circ = circularity; MaxFeret = maximum Feret's diameter; MinFeret = minimum Feret's diameter; Perim = perimeter; Round = roundness; Solid = solidity.

Table S3. The results of k-means clustering[a].

	Cluster-1	Cluster-2	Cluster-3	Cluster-4	Cluster-5	Cluster-6
**P**	16,307	8,258	10,366	392	2,524	21,519
**CPZ**	4,633	8,190	1,650	3,605	2,039	7,522
**CFIX**	2,160	5,742	944	2,999	2,126	5,244
**AMK**	7,172	4,522	3,970	1,927	2,001	7,834
**NM**	5,038	4,572	2,790	1,662	1,729	6,485
**DOXY**	3,792	4,610	2,205	699	2,348	6,508
**CP**	2,546	5,965	1,620	4,228	4,253	5,854
**AZM**	7,540	3,645	4,184	28	303	8,290
**TP**	7,568	4,257	3,966	61	420	8,399
**ENX**	1,142	3,021	1,373	5,661	4,057	2,580
**CPFX**	2,468	4,770	1,671	4,336	2,298	3,905

[a]The number of cells in each cluster classified by k-means clustering is displayed. AMK = Amikacin; AZM = Azithromycin; CFIX = Cefixime; CP = Chloramphenicol; CPFX = Ciprofloxacin; CPZ = Cefoperazone; DOXY = Doxycycline; ENX = Enoxacin; NM = Neomycin; P = Parental strain; TP = Trimethoprim.

Table S4. Number of genes in each of the 19 modules obtained using a weighted gene correlation network analysis[a].

Module	Number of genes
black	145
blue	218
brown	208
cyan	59
green	165
greenyellow	101
gray	28
grey60	45
lightcyan	46
lightgreen	45
lightyellow	36
magenta	129
midnightblue	229
pink	137
purple	110
red	155
salmon	61
tan	77
turquoise	835

[a]The number of genes in the indicated module is shown.

Table S5. Lists of genes in each of six gene modules highly correlated with the morphological features measured in this study[a].

Module	Genes											
greenyellow (Major, MaxFeret, AR)	acnB	yadR	yahH	yaiB	phoA	yajB	ylaC	rna	gltA	sdhC	sdhD	sdhA
	sdhB	sucA	sucB	sucC	sucD	rhlE	ybiJ	ompX	ybjT	ihfB	ssuB	ycbW
	ycfR	icd	icdC	ychF	cysB	pspE	aldA	ydcI	fumA	nemA	ydiK	yeaC
	yeaA	edd	yedV	yedW	yeeN	mglA	mglB	mqo	folX	yfcH	argT	yfcX
	maeB	ndk	hcaR	ygaV	ygaW	syd	ygdK	yqeF	mdh	yrfG	pck	livJ
	rpoH	yhhJ	rbbA	yhiI	dctA	lldP	lldR	lldD	atpC	atpD	atpG	atpA
	atpH	atpF	atpE	atpB	atpI	rrsC	argX	hisR	leuT	proM	yigI	metR
	fadA	fadB	glpK	glpF	yiiU	sthA	rplK	rplA	yjbA	actP	yjcH	acs
	yjfP	pyrL	leuV	leuP	leuQ							
gray (Area)	rrsH	ssuE	pspB	ydeO	eda	hisG	hisD	hisC	hisB	hisH	hisA	hisF
	hisI	rcsC	emrY	emrK	yfdE	yfdV	oxc	frc	yfdX	ypdI	ssrA	kduD
	kduI	arcB	kdgK	yjgK								
lightgreen (Minor, MinFeret)	yaaY	lpxC	dgt	panE	ybaO	mdlA	mdlB	acrB	acrA	acrR	rnk	lipA
	fur	fldA	seqA	ssuA	pqiA	pqiB	ymbA	mdtG	ribA	nhoA	fumC	zwf
	nfo	yeiI	ypeB	ligA	fldB	ygfZ	yggX	mltC	kdsC	yicM	yieP	frvX
	sodA	kdgT	fpr	soxS	soxR	ryjA	yjjW	yjjI	rob			
magenta (Circ, Round)	speD	speE	cueO	pepD	betT	yaiA	clpP	clpX	hupB	ybaM	priC	ybaL
	ybaQ	ppiB	holA	ybfP	ybgC	modA	modB	modC	moaA	moaB	moaC	moaD
	moaE	ybiC	mntR	yliJ	ycaK	pncB	csgA	solA	yceP	nagZ	ycgK	ycgL
	ycgM	cvrA	yddG	ydeW	ribC	rplT	chbB	prc	proQ	yebA	znuA	znuC
	znuB	yecF	yodA	yodB	amn	yeeY	sanA	fruA	fruK	ccmE	ccmC	yfdY
	yfdZ	pdxK	ypfI	ypfJ	acpS	pdxJ	pssA	gabT	proV	proW	proX	yqcC
	fucU	gcvA	ssrS	yggD	yggH	hybG	hybF	hybE	hybD	hybC	hybB	hybA
	hybO	yghW	ygiE	ygiH	ygjF	uxaA	uxaC	yraL	yrbK	yhbN	yhbG	rpoN
	yhbH	ptsN	yhbJ	npr	yrbL	yhdP	yhdA	smg	fmt	rsmB	trkA	yhdL
	yheL	yheM	yheN	yheO	trpS	gph	rpe	nudE	glpE	uspA	cysE	yibQ
	yicC	yifE	yifK	oxyS	fklB	cycA	ytfG	uxuA	uxuB			
midnightblue (Solid)	yaaA	talB	mog	yaaH	rpsT	ribF	ileS	lspA	fkpB	ispH	apaH	apaG
	ksgA	pdxA	surA	imp	djlA	rluA	hepA	sgrS	setA	leuD	leuC	leuB
	leuA	mraZ	mraW	ftsL	ftsI	murE	murF	mraY	murD	ftsW	murG	murC
	ddlB	ftsQ	ftsA	ftsZ	secM	secA	yacG	yacF	coaE	nadC	yacL	yacC
	hpt	panD	panC	pcnB	yadB	sfsA	ligT	hrpB	mrcB	yadS	btuF	pfs
	dapD	map	t44	rpsB	tsf	pyrH	frr	dxr	ispU	cdsA	yaeL	yaeT

	fabZ	*lpxA*	*lpxB*	*rnhB*	*dnaE*	*accA*	*yaeQ*	*yaeJ*	*proS*	*yaeB*	*rcsF*	*gmhB*
	codB	*codA*	*yaiE*	*phoB*	*glnK*	*amtB*	*ushA*	*ybdM*	*tatE*	*nadA*	*pnuC*	*ybiB*
	grxA	*ftsK*	*ompF*	*helD*	*putP*	*pyrC*	*ndh*	*sohB*	*tehB*	*ryeB*	*nac*	*dld*
	yeiG	*yeiE*	*ypdA*	*sseA*	*hmp*	*rrlG*	*pheA*	*rplS*	*ftsB*	*ygbE*	*cysC*	*cysN*
	cysD	*eno*	*pheV*	*metC*	*mtr*	*rpmA*	*rplU*	*yrdA*	*rplQ*	*rplB*	*rplC*	*yhjE*
	rfaL	*phoU*	*pstB*	*pstA*	*pstC*	*pstS*	*sbp*	*rpmE*	*purD*	*purH*	*aceB*	*aceA*
	aceK	*metH*	*yjbB*	*yjbC*	*yjbD*	*xylE*	*ubiC*	*ubiA*	*dgkA*	*zur*	*yjbN*	*yjbO*
	dnaB	*alr*	*ssb*	*yjcO*	*rpiR*	*yjdP*	*adiC*	*dcuR*	*dcuS*	*pheU*	*dipZ*	*cutA*
	yjeI	*yjeK*	*efp*	*ecnA*	*sugE*	*ampC*	*poxA*	*yjeP*	*psd*	*rsgA*	*orn*	*yjeS*
	amiB	*mutL*	*yjeT*	*purA*	*yjeB*	*rnr*	*rlmB*	*priB*	*rpsR*	*rplI*	*ytfB*	*ytfJ*
	ytfL	*ytfM*	*ytfN*	*ytfP*	*yzfA*	*ppa*	*mpl*	*yjgA*	*pmbA*	*cybC*	*treR*	*yjgF*
	yjgD	*valS*	*holC*	*yjgP*	*yjgQ*	*yjjA*	*dnaC*	*dnaT*	*rsmC*	*holD*	*rimI*	*yjjG*
	prfC	*lplA*	*ytjB*	*serB*	*radA*	*nadR*	*yjjK*	*trpR*	*yjjX*	*ytjC*	*creC*	*creD*
	lasT											
**pink (Perim)**	*thrA*	*thrB*	*thrC*	*yaaX*	*dapB*	*cdaR*	*cstA*	*ybdD*	*rihA*	*kdpD*	*kdpC*	*kdpB*
	kdpA	*kdpF*	*cspD*	*clpS*	*clpA*	*serW*	*serX*	*ptsG*	*ompW*	*trpA*	*trpB*	*trpC*
	trpD	*trpE*	*rimL*	*ydeN*	*yneE*	*ydeA*	*dcp*	*ynfP*	*rspB*	*rspA*	*dgsA*	*pntB*
	pntA	*add*	*ydhP*	*ydhR*	*rydB*	*ydiH*	*yniB*	*yeaD*	*mipA*	*yeaT*	*manX*	*manY*
	manZ	*yobD*	*yebK*	*yecJ*	*IS092*	*yedJ*	*yedR*	*yehT*	*yehU*	*cdd*	*lysP*	*micF*
	rcsD	*glpQ*	*glpT*	*glpA*	*glpB*	*glpC*	*yfaY*	*yfbQ*	*yfbV*	*tyrA*	*aroF*	*csrA*
	rygA	*rygB*	*lysA*	*argO*	*yghZ*	*rpoD*	*sstT*	*argG*	*ryhA*	*nanA*	*yhdV*	*rplV*
	rpsS	*rplD*	*yhfA*	*crp*	*yhfK*	*tsgA*	*malQ*	*malP*	*malT*	*glgP*	*glgA*	*glgC*
	glgX	*glgB*	*asd*	*yiaD*	*uhpC*	*uhpB*	*uhpA*	*ilvN*	*ilvB*	*ivbL*	*asnA*	*rbsD*
	rbsA	*rbsC*	*rbsB*	*rbsK*	*ilvC*	*glpX*	*cytR*	*argC*	*argB*	*rrsB*	*rrlB*	*pepE*
	lysC	*malG*	*malF*	*malE*	*malK*	*lamB*	*malM*	*yjcB*	*yjcE*	*proP*	*melR*	*aspA*
	cpdB	*treC*	*treB*	*pyrI*	*pyrB*							

[a]Genes appearing in the modules that are most highly correlated with the corresponding morphological features are listed in ascending order of the gene annotation number (Blattner et al., 1997). AR = aspect ratio; Circ = circularity; MaxFeret = maximum Feret's diameter; MinFeret = minimum Feret's diameter; Perim = perimeter; Round = roundness; Solid = solidity.

Table S6. Number of patches used for patch classification[a].

Fold	Strain	Training	Test	Validation
1	**P**	20,225	12,254	4,235
	**CPZ**	9,327	4,360	2,482
	**CFIX**	6,222	4,383	1,606
	**AMK**	8,248	5,830	2,210
	**NM**	8,317	3,668	2,077
	**DOXY**	6,263	5,076	1,534
	**CP**	8,403	4,183	2,185
	**AZM**	7,431	5,536	2,032
	**TP**	7,698	5,646	1,976
	**ENX**	7,045	2,803	1,621
	**CPFX**	6,718	4,535	1,671
2	**P**	19,802	12,564	4,348
	**CPZ**	7,307	7,085	1,777
	**CFIX**	6,787	3,602	1,822
	**AMK**	9,718	3,992	2,578
	**NM**	6,128	6,272	1,662
	**DOXY**	7,516	3,632	1,725
	**CP**	7,608	5,185	1,978
	**AZM**	8,204	4,455	2,340
	**TP**	8,710	4,408	2,202
	**ENX**	5,800	4,255	1,414
	**CPFX**	7,265	3,764	1,895
3	**P**	20,453	11,896	4,365
	**CPZ**	9,218	4,724	2,227
	**CFIX**	6,423	4,226	1,562
	**AMK**	7,776	6,466	2,046
	**NM**	7,815	4,122	2,125
	**DOXY**	7,041	4,165	1,667
	**CP**	7,573	5,403	1,795
	**AZM**	7,965	5,008	2,026
	**TP**	7,964	5,266	2,090
	**ENX**	5,729	4,411	1,329
	**CPFX**	6,621	4,625	1,678

[a]The number of patches extracted from the microscopy images and used for training, test, and validation sets are shown. AMK = Amikacin; AZM = Azithromycin; CFIX = Cefixime; CP = Chloramphenicol; CPFX = Ciprofloxacin; CPZ = Cefoperazone; DOXY = Doxycycline; ENX = Enoxacin; NM = Neomycin; P = Parental strain; TP = Trimethoprim.

Table S7. Number of cells used for single-cell classification[a].

Fold	Strain	Training	Test	Validation
1	**P**	31,556	19,664	8,146
	**CPZ**	16,340	6,848	4,451
	**CFIX**	9,750	6,839	2,626
	**AMK**	13,592	10,250	3,584
	**NM**	13,449	5,439	3,388
	**DOXY**	9,749	8,052	2,361
	**CP**	14,145	6,665	3,656
	**AZM**	11,662	9,004	3,324
	**TP**	11,916	9,583	3,172
	**ENX**	11,135	4,186	2,513
	**CPFX**	10,167	6,784	2,497
2	**P**	30,375	20,765	8,226
	**CPZ**	11,748	13,063	2,828
	**CFIX**	10,474	5,874	2,867
	**AMK**	17,023	5,845	4,558
	**NM**	9,066	10,775	2,435
	**DOXY**	12,115	5,440	2,607
	**CP**	12,400	8,786	3,280
	**AZM**	13,084	6,953	3,953
	**TP**	14,410	6,581	3,680
	**ENX**	9,021	6,608	2,205
	**CPFX**	10,830	5,808	2,810
3	**P**	32,195	18,937	8,234
	**CPZ**	15,982	7,728	3,929
	**CFIX**	10,204	6,502	2,509
	**AMK**	12,743	11,331	3,352
	**NM**	12,753	6,062	3,461
	**DOXY**	11,022	6,670	2,470
	**CP**	12,541	9,015	2,910
	**AZM**	12,666	8,033	3,291
	**TP**	12,758	8,507	3,406
	**ENX**	8,838	7,040	1,956
	**CPFX**	10,099	6,856	2,493

[a]The number of cells segmented from microscopy images and used for training, test, and validation sets are shown. AMK = Amikacin; AZM = Azithromycin; CFIX = Cefixime; CP = Chloramphenicol; CPFX = Ciprofloxacin; CPZ = Cefoperazone; DOXY = Doxycycline; ENX = Enoxacin; NM = Neomycin; P = Parental strain; TP = Trimethoprim.

Table S8. Genes significantly different in their expression levels between each resistant strain and the parental strain and highly correlated with morphological features[a].

Strain	Features	Genes		
**CPZ**	Perim	*malE*		
	Solid	*ompF*		
**CFIX**	Solid	*ompF*		
**AMK**	-			
**NM**	Major/MaxFeret/AR	*mglA*	*mglB*	*ygaW*
	Solid	*ompF*		
**DOXY**	Perim	*cstA*	*glpT*	*malE*
	Major/MaxFeret/AR	*mglB*		
	Solid	*ompF*		
**CP**	Solid	*ompF*		
**TP**	Circ/Round	*yodA*		
**AZM**	-			
**ENX**	Solid	*ompF*		
**CPFX[b]**	-			

[a]The top 100 genes with the largest absolute differences in expression between each resistant strain and the parental strain were selected from the transcriptome data (Suzuki et al., 2014). Genes common to all four lines of each resistant strain were retained, and those highly correlated with the morphological features calculated by WGCNA were identified. [b]Frameshift mutations in *ompF* occur in the CPFX-1, CPFX-2 and CPFX-4 strains, while significant repression of *ompF* gene expression is observed in the CPFX-3 strain (Suzuki et al., 2014). AMK = Amikacin; AZM = Azithromycin; CFIX = Cefixime; CP = Chloramphenicol; CPFX = Ciprofloxacin; CPZ = Cefoperazone; DOXY = Doxycycline; ENX = Enoxacin; NM = Neomycin; TP = Trimethoprim. AR = aspect ratio; Circ = circularity; MaxFeret = maximun Feret's diameter; Perim = perimeter; Round = roundness; Solid = solidity.
